# Silicon-28-Tetrafluoride as an Educt of Isotope-Engineered Silicon Compounds and Bulk Materials for Quantum Systems

**DOI:** 10.3390/molecules29174222

**Published:** 2024-09-05

**Authors:** Owen C. Ernst, David Uebel, Roman Brendler, Konstantin Kraushaar, Max Steudel, Jörg Acker, Edwin Kroke

**Affiliations:** 1 Institut für Materialchemie, Brandenburgische Technische Universität Cottbus-Senftenberg, 01968 Senftenberg, Germany; owen.ernst@b-tu.de (O.C.E.); uebel@b-tu.de (D.U.); roman.brendler@b-tu.de (R.B.); joerg.acker@b-tu.de (J.A.); 2 Institut für Anorganische Chemie, TU Bergakademie Freiberg, 09599 Freiberg, Germany; konstantin.kraushaar@chemie.tu-freiberg.de (K.K.); max.steudel@student.tu-freiberg.de (M.S.); 3 Zentrum für Effiziente Hochtemperaturstoffwandlung (ZeHS), TU Bergakademie Freiberg, 09599 Freiberg, Germany

**Keywords:** isotopes, quantum computing, spintronic, microelectronics, enrichment, isotope engineering

## Abstract

This review provides a summary of the existing literature on a crucial raw material for the production of isotopically pure semiconductors, which are essential for the development of second-generation quantum systems. Silicon-28-tetrafluoride (^28^SiF_4_) is used as an educt for several isotope-engineered chemicals, such as silane-28 (^28^SiH_4_) and silicon-28-trichloride (^28^SiHCl_3_), which are needed in the pursuit of various quantum technologies. We are exploring the entire chain from the synthesis of ^28^SiF_4_ to quantum applications. This includes the chemical properties of SiF_4_, isotopic enrichment, conversion to silanes, conversion to bulk ^28^Si and thin films, the physical properties of ^28^Si (spin neutrality, thermal conductivity, optical properties), and the applications in quantum computing, photonics, and quantum sensing techniques.

## 1. Introduction

Fluorine is monoisotopic, which makes its gaseous compounds well-suited for isotopic enrichment. A popular example is the enrichment of stable, non-radioactive isotopes. This review covers the enrichment, chemical properties, conversion, and application of silicon-28 tetrafluoride ^28^SiF_4_. 

For a considerable period of time, the study of isotopically pure semiconductors was a minor area of interest within the fields of chemistry, physics, and materials science. It is known that the mass of the atomic nucleus can alter the properties of a molecule. However, this effect was considered to be negligible for the majority of properties and for most elements heavier than hydrogen. It is now understood that these seemingly minor changes are of significant importance in microelectronics, metrology, and quantum system technology. In particular, the latter has led to a rapid increase in demand for isotopically pure semiconductors, most notably ^28^Si. 

The development and production of second-generation quantum systems, including semiconductor quantum computer chips and quantum sensors, is contingent upon the availability of ^28^Si [1,2]. These quantum computers are based on the use of trapped electrons as quantum bits (qubits) whose spin is utilized. The main advantages of ^28^Si can be attributed to three central properties:

(i) Spin vacuum: The coherence of the electron spin states is disrupted by the interference of nuclear spins, for instance, from ^29^Si nuclei, which results in the loss of functionality of the qubits [3]. Silicon crystals made of nuclear-spin-free stable isotopes like ^28^Si and ^30^Si are ideal for hosting spin quantum bits due to their coherent properties, which are not disrupted by host nuclear spins. The property known as spin vacuum results in sharp ensemble donor resonances and extended spin relaxation times [4]. In pure ^28^Si material, the theoretical maximum for qubit decoherence times is 10 h [5]. In addition to quantum computing, the ^28^Si spin vacuum is also employed to stabilize optically active quantum dots in silicon [6].
(ii) High thermal conductivity: The phonon scattering and, consequently, the thermal conductivity are markedly changed at low temperatures in the isotopically pure materials [4,7]. The formation of scattering modes is primarily caused by the presence of impurities, doping agents, defects, and the natural mass distribution of isotopes. It is possible to reduce the impact of the former factors by optimizing the crystal growth and deposition processes. The thermal conductivity of high-purity, monocrystalline, low-defect ^nat^Si with a natural isotope distribution is 45 Wcm^−1^K^−1^ at a temperature of 21 K. With the use of isotopically pure ^28^Si, the thermal conductivity at 21 K can be increased to 450 Wcm^−1^K^−1^ when the material is 99.99% pure. This value exceeds the previous maximum conductivity of ^12^C diamond at 104 K of 410 Wcm^−1^K^−1^. Consequently, the highest thermal conductivity ever measured for a dielectric was achieved [7].
(iii) Sharp spectroscopic states: The analysis of photoluminescence spectra indicates that the homogeneous mass of the ^28^Si atoms in the crystal lattice results in a shift of the band gap by 58 meV [8]. Furthermore, high-energy measurements reveal a more concise fine structure of the non-phononic photoluminescence lines of the bound excitons. The optimized fine structure in the photoluminescence spectrum can be used for excitonic processes in isotopically pure materials with high resolution, for example, for quantum sensing.

The first steps from the laboratory to technical application are currently being discussed for some second-generation quantum systems [9]. However, a significant proportion of the technologies in question remain at the conceptual stage, with few tangible outcomes. One reason for this is that the required ^28^Si is the end product of a complex value chain, which makes it challenging to obtain. Furthermore, there is a lack of well-functioning, stable production routes that can facilitate the necessary processes. 

This review paper follows the necessary technical steps to precure ^28^Si in its applicable forms, in particular ^28^SiH_4_ and bulk ^28^Si, from ^28^SiF_4_. 

It is not possible to enrich elemental silicon on an industrial scale. Even monosilane (SiH_4_), which is frequently used for Si synthesis in industry, is unsuitable for this purpose. Enrichment occurs almost exclusively with silicon tetrafluoride (SiF_4_). Since ^28^SiF_4_ has only been used for research purposes so far, there are no data on the price development of the enriched gas. However, due to the high energy input required for enrichment, prices can be expected to be in the order of EUR 100,000 per kg for a purity of 99.9%. Each additional order of magnitude of isotopic purity can increase the price significantly. Similarly, the conversion of SiF_4_ to SiH_4_ or Si is anticipated to elevate the price by a factor of 2–10. 

Its high price is attributable to the great technical effort involved in its manufacture. The only commercially viable method is its enrichment by a magnitude of centrifuges. These use the counter-current centrifuge principle. The method described in the paper offers only a low separation performance per center joint, which requires a large number in a cascade. 

For this reason, there have always been competing approaches to enrichment, some of which are still being pursued: thermal diffusion, targeted excitation of individual isotopes using laser radiation (also known as the SILEX process) or microwaves, as well as the separation of isotopes using cyclotrons. As we describe later, these methods often provide unsatisfactory results.

The prospective commercialization of selected quantum technologies is contingent upon the availability of ^28^SiF_4_ as a pivotal precursor material. It is necessary to prepare isotopically pure SiF_4_ for material synthesis to improve silicon’s physical properties, especially in the context of quantum technology development [10]. This highlights the critical role of isotopically pure silicon tetrafluoride in advancing cutting-edge technologies. 

Historically, further processing of ^28^SiF_4_ has been a challenge, which often only was solved by international cooperation. Also, up to this day, no stable and commercially viable method has been introduced. The earliest reported source mentioning pure ^28^Si was an American experiment, which was vague about its origin [11]. Unfortunately, no details are given as to how it was produced. The enrichment was probably carried out using a cyclotron, and from there, the simplest possible further processing was used: ^28^SiO_2_ was produced by combustion, which was then reduced with Al powder [12]. When isotope-pure silicon regained interest at the beginning of the 1990s, this process was taken up again. However, there were disadvantages: high losses, heavy contamination with Al, and only bulk ^28^Si as a product. Alternative routes that produced ^28^SiH_4_ as a final or intermediate product were introduced: H-substitution with metal hydrides such as CaH_2_, LiAlH_4_, and NaH. 

In this article, we present the first comprehensive overview of this crucial isotopically pure material. We examine ^28^SiF_4_ in depth, exploring its synthesis, properties, enrichment, further processing, and applications. 

## 2. Chemical Properties and Processing of ^28^SiF_4_

### 2.1. Synthesis and Selected Properties of SiF_4_ (and ^28^SiF_4_)

**Figure 1:**
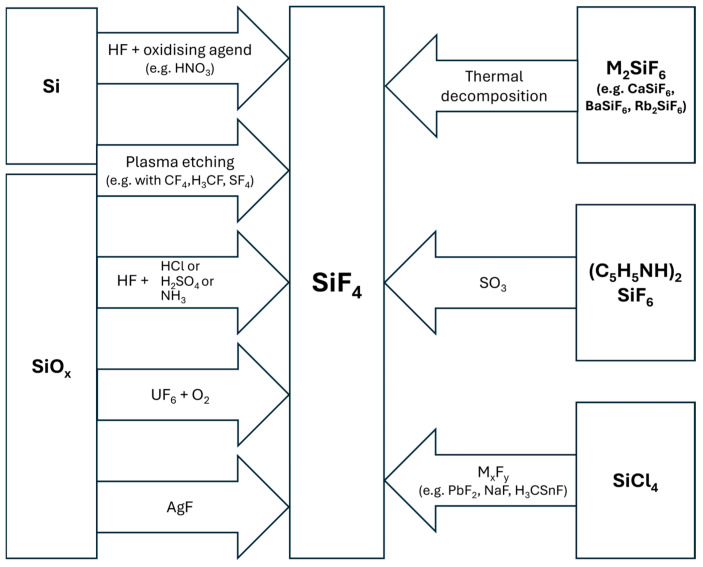
Summarized reaction pathways of the known synthesis routes to SiF_4_.

The synthesis procedures of silicon tetrafluoride are typically conducted with chemicals that are not isotopically enriched. Furthermore, all chemical reactions leading to SiF_4_ would proceed in a similar manner with educts containing any of the stable isotopes ^28^Si, ^29^Si, and ^30^Si. This is due to the small, relatively insignificant differences in molecular masses. In contrast, reactions involving hydrogen (or deuterium) exhibit significant differences in reaction rates and, consequently, product yields [13]. Nevertheless, non-mass-dependent isotope effects are known to be important for selected properties (see Section 2.1.3 and Section 2.1.4) [13]. However, they do not play a role in the chemical synthesis routes to prepare SiF_4_. In the following picture, a synthesis workflow is depicted (Figure 1).

#### 2.1.1. Synthesis Routes and Formation of SiF_4_

Few review articles on the general laboratory preparation and industrial chemical synthesis of SiF_4_ have been published. While there are brief overviews available, to the best of our knowledge, all of them are in Chinese [14,15,16] or in Russian [17,18]. Consequently, it seems prudent to provide a compilation of the published synthesis routes to SiF_4_ in this review. 

The earliest reports known on the synthesis and properties of SiF_4_ date back to the late 18th and early 19th centuries. Chemists such as H. Davy [19] and others described the reaction of fluorspar (CaF_2_) (1) with sulfuric acid in the presence of glass powder or in glassware, yielding gaseous products that most likely consisted mainly of SiF_4_ (2). For reaction (2), the reaction enthalpy is −137.11 kJ/mol [20].

CaF_2_ + H_2_SO_4_ → 2 HF + CaSO_4_ (1)

4 HF + SiO_2_ → SiF_4_ + H_2_O (2)

This synthesis also works reasonably well with metal silicates and other SiO_x_-containing substances, provided that a sufficient excess of sulfuric acid is utilized to prevent the silicon fluoride from being hydrolyzed. The purity of the obtained product varies depending on the purity of the starting materials and other reaction parameters. 

Significant quantities of SiF_4_ are generated as a byproduct in the production of phosphate fertilizers. This occurs when fluorapatite Ca_5_(PO_4_)_3_F and/or CaF_2_ contained in phosphate raw materials are treated together with SiO_2_ or silicate minerals. It is common for phosphate rock to contain about 3–4% fluoride [21]. During the process, the fluorine present in the rocks is predominantly converted to hexafluoro silicic acid H_2_SiF_6_. The latter is formed from a further reaction of HF with SiF_4_. The majority of the H_2_SiF_6_ is used in the production of AlF_3_. However, it can also be used to reproduce SiF_4_ and HF upon heating (see below).

The direct preparation of SiF_4_ from the elements is possible. It is highly exothermic with a reaction enthalpy of -1615 kJ/mol (see Table 1). Thus, if no precautions are taken, a reaction at room temperature can cause ignition and inflammation. However, fluorination of elemental silicon at −70 °C in freon-11 yields SiF_4_ (3) in a smooth and immediate reaction (reaction enthalpy −1615.78 kJ/mol) [22,23].

Si + 2 F_2_ → SiF_4_ (3)

Binary fluorides may be employed as fluorinating agents in order to exclusively obtain SiF_4_, even in high purity. Uranium fluorides UF_6_ and UF_4_ are examples. In the latter case, high-purity SiO_2_ reacted in air at elevated temperatures of 400–800 °C in accordance with the following equation [24]:

3 UF_4_(s) + 3 SiO_2_(s) + O_2_(g) → U_3_O_8_(s) + 3 SiF_4_(g) (4)

Solid silicon monoxide, SiO, reacts at 450 °C with silver fluoride, AgF, to give SiF_4_ [25]:

2 SiO + 4 AgF → SiF_4_ + SiO_2_ + 4 Ag (5)

A standard and well-investigated laboratory process for the production of SiF_4_ is the thermal decomposition of metal hexafluoro silicates M_2_(SiF_6_) with M = alkali metal (Li, Na, K, Rb, Cs), e.g., sodium [26], or M(SiF_6_) with M = alkaline earth (Ca, Sr, Ba) metal, etc. J. Zachara and W. Wigniewski conducted a thermolysis study and found the expected reaction behavior for Li_2_SiF_6_, Na_2_SiF_6_, CaSiF_6_, SrSiF_6_, and BaSiF_6_ (reaction enthalpy −2970 kJ/mol at 655 K [27]) predicted by the following equations [28]:

M_2_SiF_6_(s) → 2MF(s) + SiF_4_(g) (6)

MSiF_6_(s) → MF_2_(s) + SiF_4_(g) (7)

For K_2_SiF_6_, Rb_2_SiF_6_, and Cs_2_SiF_6_, they found the following:

3 M_2_SiF_6_(s) → 2 M_3_SiF_7_(s) + SiF_4_(g) (8)

The temperature of maximum mass loss during the decomposition of the investigated fluorosilicates under investigation exhibited a clear correlation, with values ranging from 300 °C for M = Ca to 810 °C for M = Rb. This correlation can be attributed to the fact that the larger the charge density on the metal cation, the lower the decomposition temperature. 

The initial metal fluorosilicates may be prepared from the corresponding metal carbonates and hydrofluoric acid or, alternatively, by precipitation if the solubility of the hexafluoro silicate is low. The presence of carbonate impurities may result in the contamination of the generated SiF_4_ gas with C1–C4 hydrocarbons [29]. 

Pyridinium hexafluoro silicate reacts already at room temperature with SO_3_ (obtained by distillation of oleum in vacuum) to give SiF_4_ [30]:

(C_5_H_5_NH)_2_SiF_6_ + 2 SO_3_ → 2 C_5_H_5_NHSO_3_F + SiF_4_ (9)

An alternative approach involved the preparation of pure SiF_4_ in 66% yield from SiCl_4_ by refluxing with PbF_2_ in MeCN [31]. Other metal fluorides, such as NaF, can be used in place of the lead salt [32]. 

A more feasible laboratory route to SiF_4_ is based on trimethyltin fluoride, a mild fluorinating agent. The solid reagent is treated with liquid SiCl_4_ at room temperature, furnishing SiF_4_ with a 97% yield. No solvent is necessary [33]:

4 Me_3_SnF(s) + SiCl_4_(l) → 4 Me_3_SnCl + SiF_4_ (10)

A metathesis reaction between SiCl_4_ and CaF_2_ can be performed at 400–500 °C to prepare SiF_4_ [34]. At slightly higher temperatures, a dry mixture of SiO_2_ and PbF_2_, mixed in stoichiometric amounts under a high vacuum, also yields SiF_4_ [35]. 

In many cases, acidic wet chemical etching and dissolution of silicon [36] involves the use of solutions containing HF to produce wafers for the photovoltaic and semiconductor industries. The resulting SiF_4_ is partially dissolved as H_2_SiF_6_ or in ionic form as [HSiF_6_]^−^ and [SiF_6_]^2−^, depending on the pH value. In order to achieve optimal reaction rates, an oxidizing reagent is typically required. In the absence of an oxidizing agent, aqueous HF solutions or HF solutions containing additional non-oxidizing components, such as sulfuric acid (H_2_SO_4_), hydrochloric acid (HCl), or ammonia (NH_3_), will dissolve materials (usually in layered forms), in which the silicon is already oxidized, such as SiO_2_, SiO_x_, SiO_x_N_y_, or SiO_x_P_y_H_z_ (phosphor silicate glass, PSG) forming SiF_4_. In the case of elemental silicon, nitric acid (HNO_3_) is often employed as the oxidizing agent. However, numerous alternative compounds can be used, including ozone (O_3_) [37], hydrogen peroxide (H_2_O_2_) [38], nitrosyl ion (NO^+^) salts [39], chlorine (Cl_2_) [40], or bromine (Br_2_) [41]. If no oxidizing agent is present, the dissolution of silicon is not observed, resulting in the formation of a Si-H surface passivation [35]. However, a recent study has reported the dissolution of silicon in a hydrofluoric acid solution at a very low rate, which may be attributed to the presence of dissolved oxygen acting as the required oxidizing agent [42]. At very low concentrations of HNO_3_, silicon is oxidized under the formation of hydrogen and nitrogen monoxide [43,44].

(11) $$\mathrm{Si}+\frac{z}{3}\;{\mathrm{HNO}}_{3}+6\;\mathrm{HF}\;\to \;H_{2}{\mathrm{SiF}}_{6}+\frac{4-z}{2}\;H_{2}+\frac{z}{3}\;\mathrm{NO}+\frac{2z}{3}\;H_{2}O\;(2\;\leq \;z\;\leq \;4)$$

In the semiconductor industry, during the formation of silicon-based micro-electro-mechanical devices, photovoltaic cells are processed, and in related industrial branches, the formation of SiF_4_ also occurs during the dry chemical etching of silicon-containing materials. An example of the latter is fluorocarbon-based plasmas, which are employed for the dry (plasma-assisted) etching of ultra-low-κ silica layers [45]. Similarly, the formation of SiF_4_ is observed when other fluorine-containing chemicals are used for dry (plasma-assisted) etching in the aforementioned areas. Examples include SF_6_ [46] and NF_3_ [47]. The most prevalent directional etching currently in use is reactive-ion etching (RIE). RIE typically employs a multitude of different etching gases, frequently containing fluorine (e.g., CF_4_, HBr, O_2_, CH_3_F, SF_6_) in a plasma mixture with high ion energies (∼1 keV) for directionality [48]. Moreover, plasma-less gas-phase cleaning [49] and, more recently, thermal atomic layer etching (ALE) [50] approaches have been developed. SiF_4_ is formed in all cases involving Si, SiC, SiO_2_, or other silicon-containing substrates. This is attributed to the extremely high thermodynamic stability and the very high volatility of SiF_4_. 

Due to the extremely high global warming potential and persistence of SF_6_ in the atmosphere, a recent proposal has been put forth to transform this species (which is used, for instance, as an insulator gas in various applications and for dry chemical etching as previously mentioned) into SiF_4_ via a process mediated by Fe_2_O_3_/Cr_2_O_3_ composites [51]. 

#### 2.1.2. Solid State, Gas Phase, and Electronic Structure of SiF_4_

The tetrahedral molecule structure of SiF_4_ has been the subject of extensive study for decades. One of the most intriguing features is its very short Si-F bond length. The solid-state molecular structure was first reported by G. Natta in 1930, who resolved powder X-ray diffraction data obtained at −170 °C [52]. He determined a value for the Si–F bond length of 156 ± 1 pm. The SiF_4_ molecules are arranged in a body-centered cubic lattice. In 1934, L. O. Brockway and F. T. Wall performed electron diffraction in the gas phase and found an atomic distance of 154 ± 1 pm [53]. Single crystal structure analyses confirmed this result. A measurement at -117 °C gave a Si-F bond length of 154.01 ± 0.06 pm (cubic space group I43m, Z = 2, a = 547.6 ± 0.1 pm) [54]. These experimental results deviated from the simple theoretical approach to calculating bond length values of molecular species from the sum of covalent atomic *radii*. In order to include ionic bonding contributions, corrected atomic *radii* values were derived [55]. An ionic bond contribution of 70% was derived from the difference in electronegativity between silicon (EN_PAULING_ = 1.9) and fluorine (EN_PAULING_ = 4.0) [56]. However, this amount of ionic character, if uncompensated, would place a very large charge of +2.8 on the silicon atom in SiF_4_. Consequently, the concept of multiple bonding or pi-back-donation was proposed by L. Pauling. This would reduce the charge on the silicon atom [35]. A 65% double-bond character in SiF_4_ would result in a positive charge value of +0.2. Furthermore, he proposed that the unusual thermal stability (see below) of SiF_4_ was due to its resonant structure and a bond order of approximately 1.5 for the four Si-F bonds in SiF_4_. However, in other approaches, for example, those of R. J. Gillespie and others, SiF_4_ and related molecules such as BF_3_ have been described as predominantly ionic molecules [57]. 

A high-resolution rotational spectroscopy investigation of ^28^SiF_4_ was conducted using infrared–microwave double-resonance spectroscopy in order to gain insight into the matter [58]. K. J. Donald et al. have published ab initio molecular orbital calculations employing a natural bond orbital population analysis to rationalize bonding patterns in halosilanes and halogermanes (MH_4−*n*_X*_n_*, *n* = 1–4; M = Si, Ge; X = F, Cl, Br). In contrast to the heavier halogens Cl and Br, the geometric and electronic properties of the fluorine compounds are unique. These observations include an *n*-independent charge density at the F atoms and a significant decrease in the M–F bond length as *n* increases [59]. In a recent theoretical study based on adaptive natural density partitioning (AdNDP) analysis, it was suggested that there are five 5c-2e back-donating bonds in addition to the four 2c-2e sigma bonds [60]. The authors propose that the synergistic back-donation and 18e rule are prevalent in pentatomic tetrahedral compounds, as well as in isoelectronic molecules and ions, such as ClO_4_^−^, SO_4_^2−^, PO_4_^3−^, and XeO_4_. 

#### 2.1.3. Physical and Chemical Properties 

The properties of chemical compounds are, in general, dependent on their isotope composition [61]. However, in most cases, the differences are very small and can be neglected. This holds also true for ^28^SiF_4_, ^29^SiF_4_, and ^30^SiF_4_, as well as for mixtures of these molecules. A summary of the most relevant physical properties of SiF_4_ based on the naturally occurring silicon isotope mixture is listed in the following Table 1.

**Table 1:** Physical data of SiF_4_.

Property	Symbol	Unit	Value	Reference
Melting point	mp	°C	−95.0 (sublm.)	[62]
Boiling point	bp	K	177.1	[63]
Critical temperature	T_C_	K	259	[64]
Critical pressure	p_C_	bar	37.1457	[64]
Vapor pressure	P°	Pa	3.36 × 10⁵ (at 190 K)	[65]
Heat capacity	C	J/molK	73.621	[66]
Ionization potential	IE	eV	15.29 ± 0.08	[67]
Bond dissociation energy (homolysis)	D_0_	kJ/mol	565	[68]
Appearance energy (heterolysis, SiF_3_^+^)	AE	eV	16.2 ± 0.1	[69]
Standard enthalpy of formation	ΔH_f_	kJ/mol	−1615 (at 298 K)	[66]
Standard entropy	S	J/molK	282.76 (at 298 K)	[66]
Viscosity	v	m^2^/s	0.404 × 10^6^ (at 300 K; 975 kPa)	[70]
Density	ρ	g/cm^3^	0.00469 (at 760 mmHg)	[71]

#### 2.1.4. Isotope-Dependent Physical/Chemical Properties 

Isotope-dependent properties are particularly noteworthy in spectroscopy, where variations in mass and nuclear characteristics significantly influence the behavior of the compound. To illustrate, the mass-dependent properties of silicon tetrafluoride are most evident in vibrational and rotational spectroscopy, as well as mass spectrometry. 

The abundance of silicon and fluorine atoms exerts a profound influence on the vibrational frequencies of SiF_4_, resulting in a shift toward lower frequencies. Heavier isotopes result in lower vibrational frequencies. For instance, the common isotopes ^28^Si and ^19^F create specific vibrational modes detectable by infrared (IR) and Raman spectroscopy [68]. 

Furthermore, rotational spectroscopy is influenced by the moment of inertia of SiF_4_. Consequently, the rotational constants are altered, resulting in a shift of the rotational spectra accordingly. These changes are of crucial importance for the identification of different isotopic compositions of SiF_4_ [72]. 

It is evident that mass spectrometry is influenced by the mass of the detected compound. Consequently, these techniques can be valuable for identifying the isotopic composition of compounds [73,74]. 

It is important to also consider the nuclear spin, which is a non-mass-dependent property and can be observed in nuclear magnetic resonance (NMR) spectroscopy. In the case of ^19^F NMR spectroscopy, the fluorine *nuclei* in silicon tetrafluoride provide a strong signal due to their 100% natural abundance and magnetic properties. In contrast, ^29^Si NMR spectroscopy can provide detailed insights into the local chemical environment of the silicon nucleus in SiF_4_. Due to its nuclear spin of ½, ^29^Si interacts with external magnetic fields. In contrast, ^28^Si does not interact with such fields due to its nuclear spin of zero [75]. These NMR techniques are essential for studying the structural and electronic properties of SiF_4_ without the direct influence of isotopic mass [76,77]. 

Furthermore, the optical and electronic properties of the substance must be discussed. In silicon tetrafluoride, isotopic variation has a less pronounced effect compared to vibrational and rotational properties. However, subtle differences can still occur. The electronic transitions observed in SiF_4_, as evidenced by UV-visible spectroscopy, are primarily determined by the electronic structure of the molecule. Isotopic substitution generally does not significantly alter these transitions, as they are more dependent on the electronic configuration than on the nuclear masses. However, isotopic effects can still lead to slight shifts or changes in the intensity of spectral lines due to subtle differences in nucleus–electron interactions [78]. 

Electronic properties, including ionization energy and electron affinity, are largely isotopically invariant. The mass of the isotopes has a minimal effect on the electronic structure, indicating that the chemical reactivity and bonding characteristics of SiF_4_ remain consistent across different isotopic compositions [79]. 

#### 2.1.5. Further Selected Properties of SiF_4_

Silicon tetrafluoride is a highly toxic and corrosive substance. Inhalation can cause severe respiratory irritation, including coughing, choking, and mucous membrane damage. High concentrations can lead to pulmonary edema, a serious condition characterized by fluid accumulation in the lungs. Prolonged exposure to lower concentrations can cause chronic respiratory issues such as bronchitis or COPD. In the presence of moisture, the substance will react and form hydrofluoric acid (HF), which can cause severe burns to the skin and eye and penetrate deep into tissues, resulting in delayed symptoms and potential bone damage [80,81]. 

It is of the utmost importance to implement the appropriate safety measures, including the use of personal protective equipment (PPE) and adequate ventilation when handling SiF_4_. It is also essential to have effective emergency protocols in place to manage any accidental exposure [82]. 

Silicon tetrafluoride does not occur in nature in a free state due to its reactivity. However, it can be formed through interactions between fluorine-containing minerals and silicon materials. In volcanic areas, fluorine-containing gases from magma can react with silicon dioxide (SiO_2_) to produce SiF_4_ [83]. 

Fluorine and silicon are both abundant in the earth’s crust. Some minerals containing fluorine include fluorite (CaF_2_) and cryolite (Na₃AlF_6_), while silicon is the second most abundant element, found in silicates and quartz (SiO_2_). The industrial production of SiF_4_ is achieved through a controlled process involving the reaction of hydrofluoric acid with silicon dioxide or silicate minerals, ensuring the safe and efficient production of the desired product [80]. 

### 2.2. Important Applications of SiF_4_

Silicon tetrafluoride is a versatile compound with a wide range of applications across various industries, particularly in microelectronics, photovoltaics (PV), and organic synthesis. Its distinctive chemical properties render it invaluable in numerous processes and technologies. 

In addition to monosilane (SiH_4_), SiF_4_ is employed as a precursor in chemical vapor deposition (CVD) processes for silicon-containing films, which are utilized in a range of microelectronic applications [84,85]. SiF_4_ offers advantages such as the elimination of silicon gas-phase nucleation and parasitic deposition during epitaxial growth [86]. Furthermore, SiF_4_ has been employed in plasma-enhanced CVD, demonstrating potential for use in optoelectronics applications [87]. Nevertheless, most well-established CVD processes and common commercial SiC and Si deposition processes are conducted with SiH_4_ rather than with SiF_4_. The higher rates and yields achieved with SiH_4_ as a precursor, coupled with the superior purity of commercially available SiH_4_ in comparison to SiF_4_, explain the preference for SiH_4_ (and other precursors) in most established CVD processes. Furthermore, SiF_4_ is a highly toxic and corrosive gas that requires careful handling and specialized equipment to ensure the safety of operators and the surrounding environment. This safety aspect must be given due consideration when opting for SiF_4_ as a precursor in CVD processes. 

Silicon tetrafluoride is a key component in a range of purification processes for silicon. One notable approach involves the conversion of impure silicon into SiF_4_, which can then be distilled to remove contaminants. The purified SiF_4_ is subsequently reduced back to silicon, resulting in highly pure silicon suitable for electronic applications. An effective purification process is essential for producing the ultra-pure silicon necessary for high-performance electronic devices and solar panels [88,89]. 

In the semiconductor industry, SiF_4_ is employed as a dopant gas for silicon ion implantation. This process entails the introduction of dopant atoms into silicon wafers with the objective of modifying their electrical properties. Silicon tetrafluoride serves as a source of silicon ions, enabling precise doping profiles, which are essential for the functionality of semiconductor devices [90,91]. 

As a potent fluorination agent, SiF_4_ is employed in processes that require the introduction of fluorine atoms into organic and inorganic compounds. This fluorination capability is essential in the production of various specialty chemicals and materials that benefit from the unique properties imparted by fluorine, such as increased stability and resistance to degradation [92,93]. 

Silicon tetrafluoride is a key source of fluorine in processes requiring controlled fluorine concentrations, such as the formation of low-κ dielectric films. These films, which have low dielectric constants, are crucial for reducing capacitive coupling in integrated circuits, thereby enhancing their speed and performance. The controlled release of fluorine from SiF_4_ ensures the formation of high-quality low-κ films with the desired properties [94,95]. 

Silicon tetrafluoride is also employed in the production of fluorosilicic acid (H_2_SiF_6_), a compound that is widely utilized in water fluoridation, the manufacturing of aluminum fluoride, and a variety of other industrial applications. The reaction of SiF_4_ with water can result in the formation of fluorosilicic acid, thereby underscoring the role of SiF_4_ in chemical manufacturing [96,97]. 

In organic synthesis, silicon tetrafluoride functions as a Lewis acid catalyst. Its ability to accept electron pairs renders it an effective compound in facilitating various chemical reactions, including those involved in the synthesis of complex organic molecules. This catalytic property is of particular value in the pharmaceutical and chemical industries, where precise and efficient chemical transformations are necessary [98,99]. 

SiF_4_ is employed as an educt in the synthesis of a vast array of silicon tetrafluoride adducts, which are compounds formed by the combination of SiF_4_ with other molecules usually containing higher coordinated (usually octahedrally, i.e., six-fold coordinated) silicon atoms [100]. These adducts exhibit a multitude of applications in chemical synthesis and materials science, thereby demonstrating the versatility of SiF_4_ as a precursor in the production of complex compounds [101,102]. 

In conclusion, silicon tetrafluoride is a crucial compound with a multitude of applications in synthesis, purification, catalysis, and material production. Its role in the microelectronics and PV industries, as well as its utility in organic synthesis and fluorine chemistry, exemplifies its significance in the advancement of technology and manufacturing processes. 

## 3. Enrichment 

For some applications, particularly in quantum systems, natural Si is insufficient, and isotopically pure ^28^Si is required. Enrichment is usually carried out with SiF_4_, as it fulfills the chemical and physical requirements of a substance to be enriched [103,104]:

(i) The vapor pressure of the substance must exceed 5^−10^ mmHg (≙ 0.7–1.3 kPa) as the majority of enrichment techniques require gaseous sources;
(ii) The substance must not react chemically with the materials of the enrichment chamber. Enrichment techniques exploit the slight differences in the physical properties of different isotopes. To exploit these properties, the working gas is introduced into enrichment chambers, which are usually made of stainless steel or special high-temperature alloys;
(iii) Most enrichment techniques exploit the mass-dependent inertia of the isotopes. For sufficient enrichment, the molecular mass of the molecules must not be less than 40 u. Higher molecular masses are usually associated with higher enrichment rates and lower energy consumption;
(iv) During the enrichment process, the working gas is usually heated either purposefully or by unwanted dissipative processes. The substance to be enriched must have a sufficiently high thermal stability;
(v) There should be no chemical reaction between the molecules of the working gas. Anion exchange or atom exchange would result in an incomplete enrichment. Any interaction between the molecules of the substance should be minimized.

Only a few other substances meet the requirements, including SiF_4_ and a few uranyl compounds. SiF_4_ is well suited as SiF_4_ is readily available and as the element fluorine is mononuclidic (^19^F, 9 protons and 10 neutrons in the nucleus). Thus, the mass distribution of SiF_4_ depends only on the mass distribution of the Si nucleus (Table 2). In contrast, the mass distribution in, e.g., SiH_4_ is more complex. In addition, SiH_4_ would not follow requirements (ii), (iii), and (v). Common to all enrichment technologies is that higher molar masses allow higher enrichment levels to be achieved. The required mass selectivity of ≤1 u represents a major technical hurdle, as industrial enrichment processes must be able to deliver several tonnes of isotopically pure material per year [105]. Only a few technologies meet these high requirements, despite the great efforts of many national and international research projects with high political support to find technical solutions for the enrichment of radioactive nuclei for civil and military purposes. 

### 3.1. Gas Centrifuge 

In gas centrifuges, isotope enrichment is achieved by employing the mass-dependent inertia of molecules. For the enrichment of uranium-235, this process has prevailed against its competitors and is the only enrichment technology of economic relevance today [107]. Isotope separation takes place in rotating cylinders. A gas, e.g., SiF_4_ or UF_6_, fed into the cylinders is subjected to permanent acceleration. The resulting inertial force F can be calculated using the simple relationship

(12) $$F=Mv^{2}r^{-1}$$

(*M*—molar mass; *v*—circulation speed; *r*—radius of cylinder). 

Modern gas centrifuges, also known as the ZIPPE or KAMENEV centrifuges, consist of tall, vertically rotating cylinders. Usually, they are made of carbon fiber-reinforced steel with a cylinder radius of 5 cm and can achieve rotational speeds of 750 ms^−1^ and beyond [108]. The separative power only depends on the rotational speed, not on the diameter of the cylinder [109]. High rotational speeds are no longer achieved by simple electric motors but by a rotating magnetic field that causes the cylinder itself to rotate. A force of up to 12,000 eVm (>1,000,000 g) acts on one molecule of SiF_4_. This is countered by a pressure gradient between the gas compressed against the cylinder wall and the vacuum created at the axis of rotation. If the pressure at the wall is too high, resulting from fast circulation speeds, the gas can condense on the walls of the centrifuge. 

**Figure 2:**
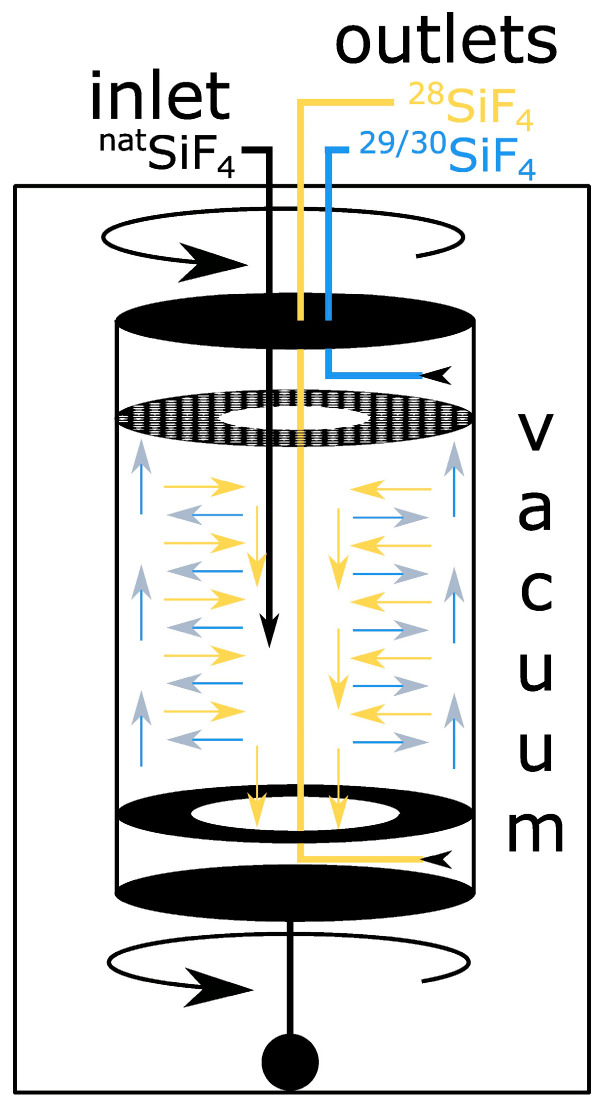
Countercurrent gas centrifuge. Natural SiF_4_ is introduced into the centrifuge. The centrifugal force generated by the rotation of the centrifuge exerts a pushing force on the heavier isotopes, causing them to move further outward than the lighter isotopes. With cylinder radii of 5–10 cm and rotation speeds of 750 m/s, the generation of forces in excess of 1,000,000 g is possible. The pressure in the regions of the centrifuge in close proximity to the wall exhibits a notable increase, whereas the pressure in the vicinity of the axis of rotation declines. In a countercurrent centrifuge, an additional flow is introduced, which results in further enrichment. By subjecting the bottom of the centrifuge to a heating process, the heavier isotopes are drawn upward, while the lighter isotopes are drawn further downward. This results in the formation of multiple vertical segments where centrifugal enrichment occurs. The heavier isotopes accumulate in the upper section of the centrifuge, subsequently traversing a perforated plate and being collected. Conversely, the lighter isotopes congregate at the base and flow through an aperture in close proximity to the axis of rotation, ultimately entering a lower chamber where they can be harvested separately.

There are several approaches to increase the degree of enrichment without increasing the rotation speed even further. The most important is the countercurrent centrifuge (Figure 2). In addition to the gas flow from the inside to the outside, a vertical pressure difference is created that pushes the molecules close to the axis of rotation downward (or upward). By heating the head of the cylinder and changing the geometry of the cylinder’s interior, a counterflow can be created that maintains or even increases the internal pressure differences. This flow results in horizontal zones within the cylinder. In each zone, horizontal enrichment occurs from the inside (light molecules) to the outside (heavy molecules). Due to the vertical pressure difference, the heavy molecules are slowly released to the zone above (or below), while the light molecules migrate to the zone below (or above). The degree of enrichment increases with each additional zone. This results in two strategies. On the one hand, many short centrifuges can be connected. The degree of enrichment can be increased by connecting the centrifuges in series, and the throughput can be increased by connecting them in parallel. This technical solution is highly flexible and can be quickly adapted to changing system parameters or product requirements. The second strategy is based on a small number of tall centrifuges. This allows many enrichment zones to be realized within one cylinder. As a result, the process runs faster and requires fewer maintenance-intensive individual components. It also minimizes contamination from cylinder walls and gas lines. The challenge with this method is the stability of the tall cylinders. When starting up the rotary motors, the bending frequencies of the cylinders must be passed. Cylinders longer than 1 m run the risk of critical deformation if they are not stabilized by specialized spring constructions. 

In 2010, the so-called Stuxnet malware spread, capable of infiltrating plant control systems. Although the origin of the computer worm has never been determined, many experts believe it was designed specifically to damage the gas centrifuges of the Iranian nuclear program by keeping the rotation frequency of the cylinders at their bending frequency [104]. 

The TC-21 centrifuges of the British company Urenco have a length of 5 m with a radius of 10 cm and rotational speeds of 770 ms^−1^ [103]. Urenco is the world’s largest producer of isotopically pure materials and relies entirely on gas centrifuges. Only the enrichment facilities of the Russian TVEL Fuel Company, a subsidiary of the state-owned Atomenergoprom group, have a similar production capacity. 

Both Urenco and TVEL have demonstrated the ability to enrich ^28^SiF_4_ to an isotopic purity of up to 99.9999%. Since Europe imposed economic sanctions on Russia, it is no longer possible to import enriched ^28^SiF_4_ from Russia to most Western countries. 

### 3.2. Laser Separation 

Since it was known that chemical isotopes have different excitation wavelengths, this property has been used to enrich isotopes. As early as the 1970s, research activities with this goal began in both the USA and Russia [110]. 

Basically, two families of techniques can be distinguished: Atomic Vapor Laser Isotope Separation (AVLIS) and Molecular Laser Isotope Separation (MLIS). In AVLIS, attempts were made to excite ^235^U directly in atomic vapor at 2500 K [111]. For this purpose, very narrow-band lasers with a wavelength of about 600 nm were developed in order not to excite other U isotopes. Several technical problems were encountered: The AVLIS systems are very susceptible to corrosion due to the high temperatures. At the same time, the throughput is low, so an excessively high uranium concentration in the gas phase leads to a transition of the excited states due to collisions between the atoms; thus, since the 1990s, only MLIS approaches have been pursued [112,113,114,115]. These use molecules such as UF_6_ or SiF_4_ as starting materials. One approach was to first excite the isotopes isotope-selectively with a laser pulse and then force them to dissociate selectively. The resulting ^235^UF_5_ could be trapped as a solid. For uranium, both pulse wavelengths are around 16 µm. This approach was also abandoned because too many impure isotopes dissociated even without excitation due to the chemical equilibrium of the system. 

**Figure 3:**
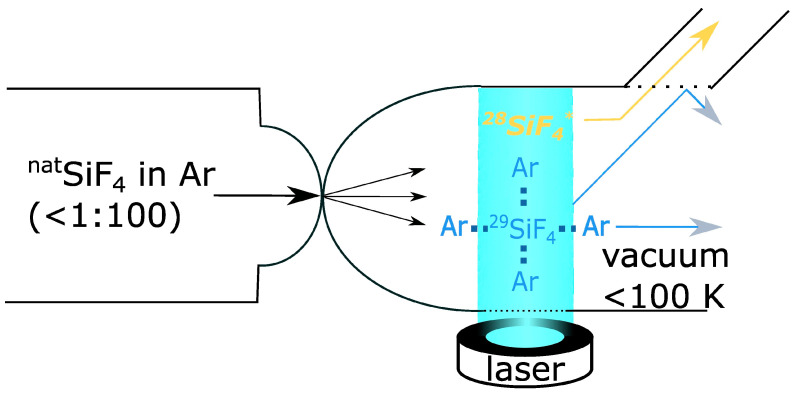
Laser separation by Silex process. The Silex process was developed by Silex System Limited and remains under the company’s operational control. A solution comprising SiF_4_ and argon is introduced into a chamber or feed pipe under conditions of increased pressure. The concentration of SiF_4_ is typically less than 1 mol%. The gas mixture is directed through a fine nozzle into a second chamber. The chamber is maintained at a constant low pressure. Because of the abrupt expansion of the gas as it traverses the nozzle, the temperature of the gas declines rapidly to a value below 100 K. At these low temperatures, the SiF_4_ molecules form stable clusters with Ar. The cold gas mixture is subsequently subjected to narrow-band laser light. The wavelength is selected to facilitate greater excitation of the ^28^Si isotope in comparison to ^29^Si or ^30^Si. Because of their excited state, the formed ^28^SiF_4_-Ar clusters disintegrate, resulting in the liberation of ^28^SiF_4_ molecules. Several techniques can be employed to separate the free ^28^SiF_4_ molecules from the larger ^29/30^SiF_4_-Ar clusters. In the simplest case, a molecular sieve can be utilized.

Only the Silex process (Figure 3) is still used today [116]. UF_6_ is highly diluted in a carrier gas and expanded through a nozzle into a vacuum chamber. The Joule-Thompson effect cools the gas to about 80 K. However, the dilution prevents the UF_6_ from condensing in the gas phase. Instead, clusters of fluoride and carrier gas particles form. After selective laser excitation of the ^235^UF_6_ species, these no longer form clusters and move through the gas phase faster than the cluster-bound UF_6_ molecules. The isotopically pure fluorides can be collected in a shielding device. 

The Australian company Silex Systems Limited has been developing the process since 1992. The process is of political relevance because this dual-use technology can, in principle, be set up in laboratories of less than 300 square meters and does not require significant energy [106]. The realization of such systems for military purposes could, therefore, go unnoticed for a long time. However, even after 30 years of development, the technology is not yet mature enough to achieve high throughput with high quality. 

Since 2019, Silex Systems Limited has been working with the University of New South Wales (UNSW) and Silicon Quantum Computing Pty Ltd. to research the production of isotopically pure ^28^SiF_4_ or ^28^Si [117]. A few grams of the material with a purity of 99.998% have already been obtained. 

On August 17, 2023, it was announced that isotope enrichment research would receive $5.1 million in funding from the Defence Trailblazer for Concept to Sovereign Capability Program, a strategic partnership between the University of Adelaide and UNSW Sydney supported by the Department of Education through the Trailblazer Universities Program. The goal is to develop and implement a production facility capable of producing up to 10 kg of enriched ^28^Si in the form of solid ^28^Si or ^28^SiF_4_ by 2027 [118,119]. 

### 3.3. Gas Diffusion 

Until 2013, large quantities of ^235^U were enriched by gas diffusion in France and the United States. Today, there is only one small plant operated by the China National Nuclear Corporation in Gansu Province. 

This family of enrichment techniques is based on Onsager’s reciprocal relations [120,121]. These state that a field or gradient can generate a force that creates a new gradient. The best-known example is the thermoelectric effect (e.g., Seebeck effect), where a temperature gradient on an electrical conductor leads to a voltage field. In isotope enrichment, on the other hand, gradient fields are used to achieve different diffusion rates for molecules of different masses. For Knudsen diffusion [122] through a pore smaller than the free path of the gas particles, it applies.

(13) $$D\approx \sqrt[{2}]{\frac{32RT}{9\pi M}r^{2}}$$

(*D*—diffusion constant; *R*—gas constant; *T*—temperature; *M*—molar mass; *r*—pore radius.) From a technical point of view, two chambers with different pressures are separated by a semi-permeable membrane [123]. The pores in the membrane only allow the Knudsen diffusion. Since the diffusion constant of the light molecules is greater, they diffuse into the low-pressure chamber faster than the heavy molecules. The degree of enrichment is further increased by a temperature gradient, which both influences the diffusion constant and allows thermal diffusion. However, the effects used are rather weak, which is why the enrichment level is 100–150 times worse than in modern gas centrifuges. For this reason, the gas diffusion process is repeated up to 1200 times until the isotopic purity is high enough for technical applications. 

As a result, the energy cost of operating a gas diffusion plant is about 50 times higher than that of a gas centrifuge. The only advantage is the much higher throughput possible with gas diffusion. Creative attempts have been made to make commercial use of the residual heat. At Pierrelatte on the Rhône, for example, a crocodile farm was opened near the reactors to use the heat for the reptiles [124,125]. Despite these efforts, all western plants were shut down by 2013 due to inefficiency. 

In principle, there is nothing to prevent the enrichment of ^28^SiF_4_ by gas diffusion. However, according to our research, no experimental tests have ever been carried out. 

### 3.4. Other and Discontinued Technologies 

The California University Cyclotron (Calutron) was an oversized mass separator used by the U.S. to enrich uranium for nuclear weapons during World War II. Ernest Lawrence developed the system based on isotope separation in a mass spectrometer [126,127,128]. Molecules are fragmented and ionized. Magnetic fields are used to force the ions into circular orbits, the radius of which depends on the ion mass. By varying the magnetic field, specific masses can be released and collected. The operation of a cyclotron is extremely energy-intensive, which is why this method has only been used on a small scale since the end of the Second World War [129,130]. The enrichment of very small amounts of ^28^Si by this method is conceivable but probably not economical. 

The subject of silicon enrichment in magnetic fields has recently been the subject of renewed interest. The silicon atoms were ionized and separated according to their mass using a cyclotron [131,132,133]. However, the ^28^Si ions (typically Si^−^ or Si^2+^) are not measured using a mass spectrometer but are implanted into a Si wafer via an ion beam. The ^28^Si ions displace the natural ^29^Si and ^30^Si atoms, resulting in an enriched ^28^Si layer in close proximity to the surface. By employing a velocity selector, also known as a Wien filter, enrichment levels of 99.9997% ^28^Si could be achieved at a depth of up to 100–200 nm [134]. 

An aerodynamic nozzle process was developed in Germany in the 1980s [135,136,137]. The goal was to find a simple alternative to gas centrifuges. A concentrated, highly accelerated gas stream was directed through a nozzle onto a narrow bend. Similar to the cylinders of centrifuges, an inertial force of varying strength acts on the gas molecules. As a result, lighter molecules follow a tighter curve than their heavier counterparts. A similar process, the vortex tube process, has been used to enrich uranium in South Africa [138,139]. However, the process requires 60 times more energy than gas centrifuges and even more than gas diffusion. This process could be a technically feasible way to enrich ^28^SiF_4_, but the energy costs would be high. 

## 4. Conversion to Silanes and Solid Silicon 

**Figure 4:**
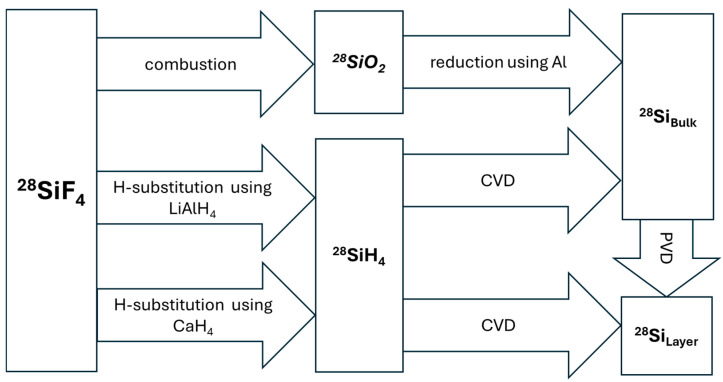
Two principal routes have been identified. The initial route entails the combustion of ^28^SiF_4_, resulting in the production of ^28^SiO_2_. This is subsequently reduced with a base metal, such as Al, to generate bulk ^28^Si. If thin ^28^Si layers are required, for example, for microelectronic applications, physical vapor deposition (PVD) methods may be employed. The second route involves the chemical substitution of fluorine atoms with hydrogen. The resulting ^28^SiH_4_ can be converted into solid ^28^Si via thermal decomposition or chemical vapor deposition (CVD).

To date, ^28^SiF_4_ has no common technical use besides its conversion into silane (^28^SiH_4_) and subsequently into solid silicon (^28^Si). The primary reason for this is that SiF_4_ has not been adopted as a standard in the chemical vapor deposition (CVD) processes of the microelectronics industry, especially in established CMOS manufacturing technologies (cf. Section 2.2). Due to the high thermodynamic stability of silicon tetrafluoride, the direct decomposition process to form elemental silicon is of low yield, highlighting the relevance of a conversion process into less stable silanes. Figure 4 gives an overview of the chemical reaction paths performed to synthesize ^28^SiH_4_ and ^28^Si from ^28^SiF_4_. 

### 4.1. Conversion to Bulk ^28^Si via ^28^SiO_2_

For a long time, the aim was to produce ^28^Si as bulk material. The main strategy was the conversion of ^28^SiF_4_ to ^28^SiO_2_ and the subsequent direct depiction of bulk material through the reduction of ^28^SiO_2_ using Al powder. The ^28^SiO_2_ powder is reduced at 1100 °C with high-purity Al powder, and the products are separated with a chemical gas transport reaction and tellurium (Te) [140].

^28^SiF_4_ + O_2_ → ^28^SiO_2_ + 2 F_2_ (14)

3 ^28^SiO_2_ + 4 Al → 3 ^28^Si + 2 Al_2_O_3_ (15)

From the literature, it can be concluded that this method or a similar approach for ^28^Si bulk material was established as early as 1958 in the USA [11]. There are no data available for the enrichment levels of these samples, and it can be suspected that the material was sourced from the Oak Ridge National Laboratory (ORNL, USA). 

The method was revived around 1994 [136] in an American–Russian–German study. ^28^SiO_2_ was sourced from ORNL and from the Russian National Metrology Institute (NMI). Notably, NMI material was discarded due to high impurities of unspecified origin, whereas ORNL material was reduced with the Al-method as described above in a collaboration between Physikalisch-Technische Bundesanstalt (PTB) and Wacker-Chemitronic in Germany. 

Czochralski (CZ) growth with a ^nat^Si seed crystal is used to produce chemically pure ^28^Si single crystals. If an ultra-pure, single-crystalline bulk material is required, the float-zone process can be used to produce a single crystal out of the polycrystalline bulk material. In this method, an inductive heater is moved slowly along the rotating polycrystalline silicon rod, forming a moving “melt zone” at the rod. Due to the thermodynamic preference of impurities in the liquid phase, an enrichment inside the melting zone is achieved while impurities accumulate at the crystal tail. This results in a purification of the solidifying silicon, which can be crystallized using a seeding crystal [141]. In 1994, crystals of 300 g exhibited notable impurities from C (1.3 × 1017 cm^−3^), O (1.1 × 1016 cm^−3^), and B (1018 cm^−3^) were obtained [136]. Another batch was sourced from the Russian Institute of Meteorology (NIM) but was not refined further due to high starting contamination. Approaches in the 1990s used ^28^SiO_2_ and resulted in contamination issues. Since the powder was reduced by Al and then sintered into a starting rod, impurities of Al and O were introduced. To circumvent these steps, a primary refinement to ^28^SiH_4_ was proposed [12]. 

### 4.2. Conversion to ^28^SiH_4_

The conversion of inert fluorides to isotopically pure semiconductor precursors presents a particular challenge. The chemical reduction of silicon tetrafluoride has been studied since the 1960s as a method to produce silane. Given the exceptionally high negative standard enthalpy of formation of silicon tetrafluoride (−1615 kJ/mol [66]), while silane has a positive standard enthalpy of formation, a significant amount of chemical energy must be applied to convert SiF_4_ to SiH_4_. This conversion requires a highly exothermic process, with an enthalpy change exceeding 2000 kJ/mol. Hydrogenation is the key strategy to convert the SiF_4_ into the thermodynamically less stable monosilane SiH_4_. 

Generally, the quantitative conversion of semimetal halides (MX_y_) to their hydrogen compounds (MH_y_) is feasible using hydrides (AH_x_).

MXy + AH_x_ → MH_y_ + AX_x_ (16)

In early research, conversions have been carried out using dissolved alkali hydrides containing aluminum (Al), lithium (Li), sodium (Na), or combinations thereof. The advantage of this method is that these hydrides can be dissolved in organic solvents like dimethyl ether, diethylene glycol, or tetrahydrofuran [12,142]. Efforts in the late 1990s to produce isotope-pure silicon utilized the reduction with lithium aluminum hydride (LiAlH_4_) or sodium aluminum hydride (NaAlH_4_). For example, LiAlH_4_ can convert SiF_4_ into SiH_4_ in diethyl ether [31].

SiF_4_ + LiAlH_4_ → SiH_4_ + LiF + AlF_3_ (17)

P. A. Lefrancois found that a slurry of sodium hydride in a high boiling solvent such as diphenyl ether can completely convert SiF_4_ into monosilane at 250 °C in under two seconds of contact time [143].

SiF_4_ + 4 NaH → SiH_4_ + 4 NaF (18)

However, the resulting fluorine salts slurry the solvent, making these methods technically very challenging to implement and scale up. Additionally, the solvent introduces organic solvents, which can act as carbon sources—electrically active donors in silicon. Additionally, metals such as aluminum are also electrically active in silicon, necessitating extensive purification. However, yields exceeding 99% have been reported [12]. 

Progress has been made with a then-novel approach using solid calcium hydride (CaH_2_) for reduction. This method’s simplicity is advantageous, as it only involves ^28^SiF_4_ and CaH_2_, avoiding the introduction of critical impurities, especially C and Al.

SiF_4_ + 2 CaH_2_ → SiH_4_ + CaF_2_ (19)

This reaction can be carried out in various ways. The solid–gas-phase reaction between SiF_4_ and CaH_2_ as a filtration combustion at temperatures lower than 300 °C was reported [144]. Higher reaction temperatures are problematic in this system due to the thermal decomposition of the formed monosilane. However, the low conversion rate due to the insufficient contact area requires several reruns of this reaction in order to obtain satisfactory yields of SiH_4_ [145]. To solve this problem, it is possible to enlarge the active surface by using milling bodies [146] or by performing the synthesis in an eutectic salt melt of KCl and LiCl with CaH_2_ solved in it [147]. The high temperatures of the salt melt (~400 °C) lead to yield losses due to thermal decomposition. A disadvantage of this synthesis is the still relatively high levels of impurities such as CH_4_, C_2_H_4_, or (SiH_3_)_2_O in the obtained silane up to 2 mol % [148,149]. Furthermore, due to the production process, the obtained silane contains calcium impurities in the ppm range, which can pose a problem for semiconductor or quantum applications [150]. 

Since the ^28^SiF_4_ used is very expensive, efficiency plays a major role—i.e., the conversion of ^28^Si from the raw material into the target material. For the CaH_2_ approach, efficiencies of up to 90% were stated [12]. However, Voltaix, an American chemical company that uses the CaH_2_ process, only achieves efficiencies of 66% [151]. 

### 4.3. Conversion to Bulk ^28^Si via ^28^SiH_4_

For bulk ^28^Si synthesis, the thermal decomposition of ^28^SiH_4_ in an adapted Siemens oven is used, with further processing by float zone refinement.

(20) SiH428 →ΔSi28 + 2 H2

The reaction conditions, such as temperature and pressure, are crucial for the quality and the structure of the grown layer, allowing the formation of polycrystalline silicon [152]. If a slim silicon rod is used as a substrate, the formation of a larger bulk rod of polycrystalline silicon is possible. This method was adapted using ^28^SiH_4_ to produce the ^28^Si bulk material for the Avogadro project [136,153]. Notably, significant quantities (5 kg) of ^28^SiH_4_ were produced beforehand using the CaH_2_ approach. 

### 4.4. Conversion to Thin ^28^Si Layers 

SiH_4_ is particularly interesting for layer deposition of thin ^28^Si films for quantum systems, which means that further processing into bulk material takes a back seat—instead, layers are used in low-pressure CVD processes. Epitaxial growth is possible at 650 °C at a pressure of 20 mmHg, allowing the formation of an epilayer of ^28^Si on a silicon wafer with natural isotopic composition. This method allows the production of epilayers with up to 300 mm in diameter [154]. 

However, more conventional technical methods would prefer halogenated silicon compounds, primarily trichlorosilane (SiHCl_3_) or tetrachlorosilane (SiCl_4_), which are commonly used in industry. Silicon bromide (SiBr_4_) is another theoretical option. Due to the high Si-F bond stability, the direct conversion of SiF_4_ into chlorosilanes or bromosilanes is not known. To date, for the deposition of ^28^Si layers, only ^28^SiH_4_ has been used as a precursor. 

Larger molecules, such as disilane (Si_2_H_6_), have no significant applications and are not discussed further. 

Another method besides CVD for the fabrication of thin silicon films on different substrates is physical vapor deposition (PVD). In this method, the material is sputtered, thermally evaporated, or plasma evaporated under a vacuum and deposited at low temperatures. This method is rather unusual for pure silicon layers [155]. 

A more specialized variant of the PVD is molecular beam epitaxy (MBE). As the name suggests, epitaxial growth of a layer on a crystalline substrate is achievable. In ultra-high vacuum conditions, the material is heated and transported as a “molecular” beam toward the also heated substrate where it decomposes. With this method, very high-purity silicon epilayers that are suitable for device applications can be achieved [156]. 

## 5. Novel Physical Properties of ^28^Si Resulting from Isotope Enrichment and Its Applications

**Figure 5:**
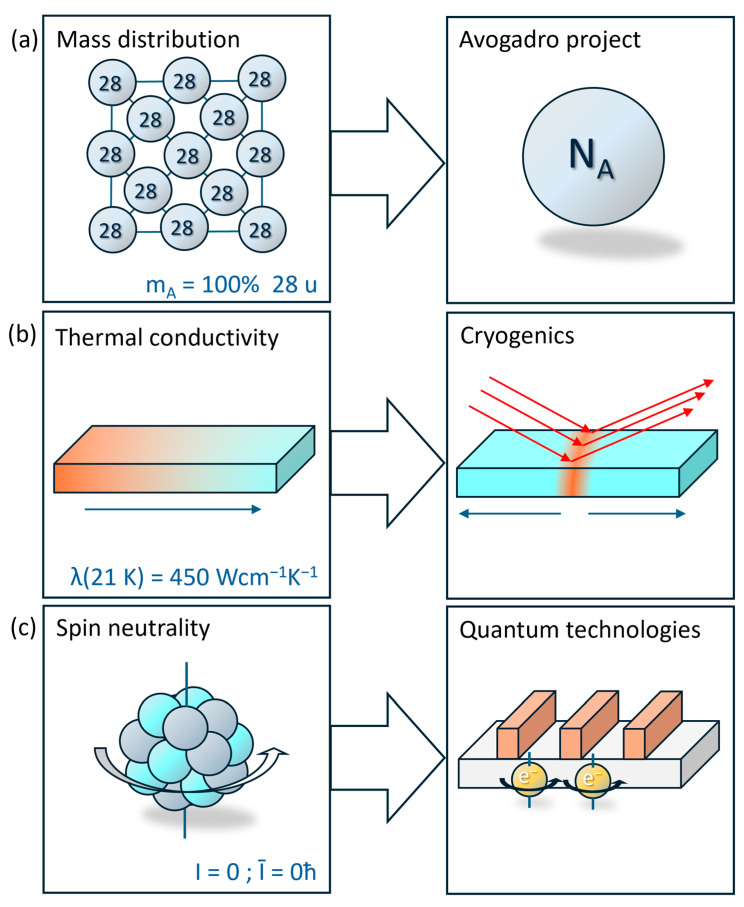
The unique properties of isotopically pure silicon have the potential to facilitate the development of novel applications. (**a**) The mass of each silicon-28 atom is precisely 28 u. This allows for the precise measurement of physical constants, such as the Avogadro constant, using a well-defined geometry, as demonstrated by the Avogadro project. (**b**) The highest heat conductivity λ of any dielectric at low temperatures has been observed in silicon-28. This offers the potential for enhanced performance in cryogenic applications, such as in high-energy laser optics in vacuum, where energy can dissipate away rapidly from laser impact, thereby reducing thermal stress or damage. (**c**) In contrast to the silicon-29 nucleus, the nucleus of silicon-28 is spin-neutral. This implies that the nucleus’ spin does not interact with the spin of electrons, thereby elongating their decoherence time and thus enabling spin quantum computer approaches.

The significance of silicon-28-fluoride in the value chain for silicon-28 single crystals and silicon-28 layers was previously highlighted. Among various silicon compounds, SiF_4_ is one of the few that meets the stringent material requirements for isotope enrichment (see Section 3). Nevertheless, the direct reduction of SiF_4_ to Si is not known. This can be attributed to the high binding energies of the Si-F bond and the unfavorable pentatomic tetrahedral configuration with unusually short bond distances, which may be due to 5c-2e bonds (see Section 2). Accordingly, the molecule must be converted to silicon-28-oxide (^28^SiO_2_) or silane-28 (^28^SiH_4_) prior to the reduction to solid silicon-28 (see Section 4). ^28^SiH_4_ has the significant advantage that it can be employed in CVD processes, which are well-established in microelectronic process technology (see Figure 3). The value chain for ^28^Si is correspondingly lengthy, with individual process steps being time-consuming and costly. Nevertheless, demand for the material is increasing. This is due to the altered properties of the isotope-pure semiconductor (Table 3 and Figure 5). 

In accordance with the established grades for semiconductor materials that are pertinent in industry, specifically ‘solar grade’ and ‘electronic grade’, ^28^Si, which is characterized by high isotopic and chemical purity, is frequently termed ‘quantum grade’ [157]. 

### 5.1. Mass Distribution|Avogadro Project 

The Avogadro project represents the most significant and extensive research project based on ^28^Si to date [158]. The fundamental concept was first proposed in 1968 [159]. It was theorized that if a perfectly round sphere of ^28^Si could be produced and its volume determined precisely, the Avogadro constant could be measured directly. The requisite conditions were the availability of precise data regarding the bond length between the ^28^Si atoms, an exceptionally high degree of chemical purity, and a crystalline structure of near-perfect quality. However, these conditions were not within the realm of technical possibility at the time. 

Starting in 2003, a further attempt was made [157]. On this occasion, a second motivation was identified in addition to the determination of the Avogadro constant. In 1999, the International Bureau of Weights and Measures (BIPM) concluded that the International System of Units (SI) should be modernized. In particular, the definition of the kilogram was deemed to be no longer appropriate. The revised definition stated that “the kilogram is the unit of mass; it is equal to the mass of the international prototype of the kilogram” [160]. The international prototype of the kilogram (IPK) has been preserved in the Pavillon de Breteuil in Sèvres, situated to the southwest of Paris, since 1889. Despite the IPK’s durability, it is gradually losing mass. It is hypothesized that the platinum–iridium (9:1) alloy is leaking hydrogen gas [161]. Over the past century, the IPK has lost a minimum of 50 µg in mass [162]. 

In order to develop a new definition, a proposal was put forth suggesting the production of a perfect sphere made of ^28^Si as one of several approaches. The objective was to create a sphere with a mass of precisely 1 kg, which could be reproduced at any time, using the Avogadro constant and the trivial mass distribution. 

Until 2019, a substantial investment in research and development was made as part of the Avogadro project, with the objective of redefining the kilogram. The Physikalisch-Technische Bundesanstalt (PTB) in Braunschweig, Germany, led a multi-stage process involving numerous international partners. The enrichment of ^28^SiF_4_ was conducted in Krasnoyarsk, Russia, while the conversion of ^28^SiF_4_ into polycrystalline ^28^Si was performed in Nizhniy Novgorod, also in Russia. Subsequently, a ^28^Si single crystal was grown using a float zone process at the Leibniz Institute for Crystal Growth (IKZ) in Berlin. In Australia, the crystal was cut and polished with the objective of achieving the most perfect spherical shape possible. The PTB assumed responsibility for the quality management of the project. The spherical interferometry method was developed at the PTB for the specific purpose of being used in the Avogadro project [163]. According to the New York Times, this is ‘one of the roundest man-made objects in the world’ [164]. 

The 144th Meter Convention on 20 May 2019 marked the redefinition of the SI units, which had been announced two decades previously. At this event, the ^28^Si sphere was not chosen as the basis for redefining the kilogram. Instead, the following is now applicable: “The kilogram, symbol kg, is the SI unit of mass. It is defined by taking the fixed numerical value of the Planck constant h to be 6.62607015 × 10^−34^ when expressed in the unit J⋅s, which is equal to kg⋅m^2^⋅s^−1^, where the meter and the second are defined in terms of *c* and Δ*ν*_Cs_” [165]. Therefore, the kilogram is determined using a Kibble balance utilizing the Josephson effect and the quantum Hall effect [166]. 

It is noteworthy that the Avogadro project, which has been operational for over a decade and a half, did yield two significant achievements. The first notable achievement was the determination of the Avogadro constant. The value of 6.02214076(12) 10^−23^ mol^−1^ determined in 2015 met all the criteria to set the constant exactly and irrevocably at this value in 2018 [167]. Another success of the Avogadro project is the first realization of a major international project with isotopically pure semiconductors. A temporary supply chain was established, and novel processes for the production, purification, and characterization of ^28^Si were devised [168,169,170,171,172,173,174,175,176]. It can be concluded that without the Avogadro project, the altered characteristics of ^28^Si would not have come into focus, and the increased demand observed in recent years (and proposed for coming years) would not have materialized. 

### 5.2. Thermal Conductivity|Cryogenics 

The thermal conductivity of silicon-28 has been found to be the highest ever recorded in a solid-state dielectric [7]. High-purity, single-crystal, low-defect ^nat^Si with a natural isotope distribution achieves a thermal conductivity of 45 Wcm^−1^K^−1^ at a temperature of 21 K. By using isotopically pure ^28^Si (99.99%), the thermal conductivity at 21 K can be increased tenfold to 450 Wcm^−1^K^−1^ and exceeds the maximum conductivity of ^12^C diamond at 104 K of 410 Wcm^−1^K^−1^. In contrast, an order of magnitude lower purity (99.92%) has a thermal conductivity of only 280 W cm^−1^K^−1^. 

The reason for the high dependence of the thermal conductivity at low temperatures on the isotopic purity can be found in the phonon scattering modes of the crystal lattice [4]. Each additional mode reduces the thermal conductivity. Scattering modes are mainly caused by impurities, doping, imperfections, defects, and the natural mass distribution of the isotopes. Former factors are minimized by crystal growth and deposition processes. Close to the minimum temperature, scattering effects of lattice distortions caused by atom mass distribution—not defects—are dominant [177]. 

The high thermal conductivity of ^28^Si makes it an attractive option for use in cryogenic technology. In particular, technologies that operate at approximately 21 K and in which heat must be dissipated can benefit from the use of the isotope-pure semiconductor. 

One illustrative example is that of X-ray optics and optical elements in synchrotron emitters. The high-energy radiation generates heat when passing through lenses and mirrors, which can result in the deformation of the components and an increase in measurement uncertainty. Some systems are already constructed from natural silicon or require Si wafers as a platform. Examples of this include micropore X-ray optics, piezoelectric actuators, and monochromators [178,179,180]. The thermal load to which the optics are exposed by the sources of the latest generation (free-electron lasers, synchrotrons) represents a significant challenge for the materials employed. The utilization of ^28^Si can enhance heat dissipation, thereby minimizing measurement inaccuracies. 

The potential utilization of ^28^Si in gravitational wave observatories is currently under discussion. A significant proportion of these large-scale physics experiments employ laser interferometry [181,182]. A laser beam is reflected between mirrors. A gravitational wave results in a slight displacement of the mirror position, which can be quantified by measuring the path difference of the laser light and the resulting interference pattern. It is essential that the mirrors possess a sufficiently substantial mass and are capable of unrestricted movement. Since 2015, gravitational waves have been detected using second-generation detectors, for example, at the Laser Interferometer Gravitational Wave Observatory (LIGO) in the USA [180]. However, the sensitivity of the measurements was insufficient for precise data analysis. This was due to two factors: seismic movements and thermal movements of the mirrors. 

Third-generation gravitational wave observatories, such as the European Einstein Telescope, are designed with the objective of reducing the aforementioned sources of noise [183,184]. The apparatus will comprise three mirrors arranged in a triangular configuration at a depth of between 200 and 300 m below the Earth’s surface. The side length of the equilateral triangle is to be 10 kilometers. Two measurement ranges are planned. The low-frequency range (1–250 Hz) will utilize a laser beam power of 18 kW, while the high-frequency range (up to 10 kHz) will employ a beam power of 3 MW. 

The seismic movements can be reduced as the seismic noise underground is less pronounced than on the Earth’s surface [185]. To minimize thermal noise, the mirrors for the low-frequency measurements are planned to be cooled to 10–20 K [182]. As the energy dissipates into the mirror surface when the laser beam is reflected, the mirror must be made of a material with high thermal conductivity. Given that the mass of the mirrors should be in the order of 100 kg, it is not feasible to manufacture them entirely from ^28^Si [186]. Currently, the production of such large quantities of the isotopically pure semiconductor is not possible. An alternative proposal is to manufacture the wire that holds the mirrors in place from ^28^Si single crystals. The first wires have already been produced in test runs at the Leibniz Institute for Crystal Growth (IKZ) in Berlin [187,188]. 

The construction of the Einstein Telescope has yet to commence. The Meuse-Rhine Euroregion (comprising Germany, Belgium, and the Netherlands), Sardinia (Italy), and Lusatia in Saxony (Germany) are under consideration as potential construction sites. The German Centre for Astrophysics (DZA) is currently being established in Lusatia, with a focus on topics such as gravitational waves [189]. Fourth-generation gravitational wave detectors are already being planned with the objective of establishing interstellar satellite laser paths of 2.5 million km or more (Laser Interferometer Space Antenna, LISA) [190]. 

Another area of interest for the low thermal conductivity of ^28^Si is low-temperature microelectronics, where an increase in the heat conductivity of the already-used silicon would enable more compact packaging. Since the effect only begins at very low temperatures, efforts in this area have been suspended; however, they could face a revival for quantum applications like quantum computing, where systems are cooled close to absolute zero anyway. This is particularly the case given that certain quantum technologies are contingent upon another property of ^28^Si, namely spin neutrality. 

### 5.3. Nuclear Spin|Quantum Technologies 

The observation of quantum effects necessitates the utilization of environments that are devoid of any form of interference. It is for this reason that quantum mechanical models and thought experiments consistently assume the existence of completely isolated particles within a closed system. In empirical experiments, these conditions can be approximated in an ultra-high vacuum. It is a contradiction that in order to realize quantum effects in technological applications, especially in microelectronics, it is necessary to stabilize the quantum states in dense solids. This presents a challenge for material synthesis and crystal growth, as non-diamagnetic defects must be avoided in the crystal lattice, as they can follow, transmit, or even amplify internal and external magnetic fields (positive magnetic susceptibility) [191,192,193]. 

Some quantum technology applications employ the spin of electrons. Their interaction is not limited to defects but also encompasses the nuclear spin present in the lattice. It is, therefore, evident that materials with nuclear spin quantum numbers ≠ 0 cannot be employed as platforms. This applies to all isotopes with an odd number of nucleons, exemplified by ^29^Si in natural silicon. Consequently, the utilization of isotopically pure ^28^Si (or ^28/30^Si) is essential for the realization of spintronic quantum technologies [3,194,195,196]. The spin-neutral environment thus created is also known as a spin vacuum. 

One example of the application of this technology is the embedding of optical emitters within silicon waveguides [6]. The objective is to generate radiating transitions in an indirect semiconductor with the aid of doping [197,198]. However, the ^29^Si nuclear spin interacts with the emitters, thereby significantly reducing the decoherence time of the radiating states. Isotopically enriched 28-silicon-on-insulator (28-SOI) chips can serve as an interference-free platform in the future and may play an important role in telecommunications in the future. 

Moreover, semiconductor quantum computers utilize the spins of electrons as the base for quantum bits (qubits), which are manipulated and measured in order to perform complex arithmetic operations. A comprehensive overview of semiconductor quantum computers can be found in the relevant literature [1,199,200,201,202,203]. 

The semiconductor quantum computer approaches can be broadly classified into two main categories: the generation of two-dimensional electron gases (2DEG) and the generation of single defects [1]. 

2DEGs can be exemplary realized in structures comprising ^28^Si and metal-oxide-semiconductor (MOS) components [204]. A 2DEG is confined at the ^28^Si/^28^SiO_2_ interface. These structures have previously been realized on 300 mm wafers using ^28^Si-CVD and have undergone a complete industry-oriented process, including lithographic preparation [2,154,205]. However, the oxidic nature of the SiO_2_ results in a high level of disorder at the interface, which represents a disadvantage of this structure [1,206]. 

As an alternative, 2DEGs can also be realized at interfaces between ^28^Si layers and silicon–germanium layers (Si_x_Ge_x−1_) layers, typically with a composition of x = 0.7 [207]. The interface disorder is less pronounced in this case. A lattice mismatch exists between the layers (^28^Si and Si_x_Ge_x−1_), resulting in a tensile strain of the ^28^Si layer. The consequence of this is an alteration in the lattice parameter, which in turn affects the electronic structure of the ^28^Si. This phenomenon is known as valley splitting [208,209]. It results in the splitting of a low-lying energy level. A second splitting occurs as a result of the application of an external magnetic field, a phenomenon known as the Zeeman effect. This results in the formation of an energetic fine structure. The spin coupling (or entanglement) of the two electrons in the levels now determines which value the qubit assumes [210]. The spins can also take on a superposition state, provided they are not subjected to any external interactions. The nuclear spin of the ^29^Si atoms would disrupt this intermediate state. It is theoretically possible for decoherence times of 10 h to be achieved in perfect ^28^Si layers [5]. ^28^Si/SiGe structures have been realized using both CVD [203] and PVD [211] processes. 

Similarly, a two-dimensional hole gas (2DHG) can be generated, wherein the role of electrons is fulfilled by holes [212]. This is not achievable in ^28^Si; instead, isotope-engineered germanium is used. 

In addition to the generation of 2DEG/2DHG, a further possibility is the generation of isolated defects. In this approach, individual atoms with nuclear spin are introduced into a spin-neutral environment (^28^Si), or a very low concentration of atoms with nuclear spin is achieved. The isolated nuclear spin generates a weak magnetic field with which individual electrons can couple, resulting in a hyperfine structure with several discrete energy levels for the electron. The electron can be read out via a radiating transition, such as a color center. 

The second method is employed, for instance, in the incorporation of phosphorus-31 atoms into ^28^Si [213,214,215]. In this process, both the electrons and the nuclear spin originate from the ^31^P. This approach is relatively challenging due to the necessity of developing industry-compatible methodologies for the targeted introduction of the ^31^P atoms. 

An alternative approach is the utilization of silicon carbide (SiC). SiC is a semiconductor that has already been employed in a number of industrial applications. 

The use of ^28^Si^12^C enables the incorporation of qubits into the color centers of the material [216,217]. This is achieved by the statistical distribution of ^29^Si atoms, which are present in a low, adjusted concentration, and their coupling with individual electrons in carbon vacancies (V_Si_^e^). It should be noted that the origin of the electron and nuclear spin differs. Nevertheless, a readable color center is also formed. The optimum ^29^Si concentration is currently estimated to be 1%. 

### 5.4. Further Selected Properties and Potential Applications 

In addition to the aforementioned properties, isotope-pure ^28^Si exhibits a number of secondary characteristics that may be of interest in specific instances or for research purposes. 

The phonon modes resulting from the isotope distribution (see Section 5.2) impact not only thermal conductivity but also neutron scattering and Raman measurements [218]. Even minimal alterations in the asymmetric mode of lattice vibrations can result in a shift of up to 10 cm^−1^ in the Raman spectra. 

Furthermore, the isotopic purity of a material can also exert an influence on photoluminescence (PL) measurements [8]. It has been possible to record the non-phononic exciton transitions of boron and phosphorus dopant atoms in ^28^Si, with a markedly sharper line width (<0.014 cm^−1^) than that observed in ^nat^Si. These highly precise measurements have enabled the fine structure in the spectrum to be resolved, which was previously obscured by the statistical isotope distribution. 

Both effects, shifting of the Raman shift and the splitting of PL peaks, can be used as characterization tools of the isotope purity of silicon and for doping control. Moreover, further insights into the excitonic processes of dopants in semiconductors could be obtained. 

A band gap shift of 58 meV can also be derived from the PL measurements [8]. As a result, the band gap of silicon can be set with great precision through isotope engineering [176]. 

It is anticipated that further insight into the phononic processes in semiconductors, and in semiconductor nanoparticles in particular, will be provided by the growth of isotopically pure Si nanowires [219]. The structures are produced via the vapor–liquid–solid (VLS) process [220]. In this variant of liquid-phase epitaxy, liquid gold droplets are deposited on a silicon substrate [221,222]. In an atmosphere saturated with silicon and an ultra-high vacuum, individual silicon atoms are dissolved in the liquid metal. Eventually, the droplet becomes supersaturated and deposits Si epitaxially at the interface with the Si substrate [223]. In principle, nanowires with isotopic gradients can also be produced in this way. One potential application of these isotopically engineered nanowires is in the creation of qubits, for example, through the targeted insertion of ^31^P atoms or through the development of all-silicon magnetic resonance quantum devices [217]. The use of isotopically modified nanowires for thermoelectrics or in microelectronics for better heat dissipation has also been discussed [224,225]. 

It can be reasonably assumed that the production of isotope-pure solar cells is a viable proposition. The enhanced thermal conductivity, in conjunction with the projected augmented carrier lifetime, should confer a distinct advantage in the domain of photovoltaics [226]. The conversion efficiency is reported to have increased by 0.7–2.5% to a maximum of 22.6% through the utilization of ^28^Si (isotopic purity: 98%) [227]. This outcome may also be attributed to the mitigation of charge carrier degradation on boron–oxygen clusters. Multiple national initiatives, including those in Japan [228], Germany [229], and Norway [230], have been undertaken with the objective of developing ^28^Si solar cells. In addition to enhancing efficiency, the empirical measurement of excitonic processes during the generation and movement of charge carriers has also been a key focus. 

The potential of ^28^Si for investigating fundamental physical phenomena and advancing technological innovation is vast. However, the material’s limited availability and the inherent complexities of its value chain are currently impeding the progress of numerous research and development initiatives. 

## Figures and Tables

**Table 2:** Comparison of the isotope distribution between SiF_4_ and SiH_4_ [106]. As hydrogen has three stable isotopes while fluorine is mononuclear, the mass distribution in SiH_4_ is more complex. For this reason, silicon enrichment processes are usually based on SiF_4_.

	Nucleon Number Si	Nucleon Number F/H	Isotopic Ratio	Molar Mass
^28^SiF_4_	28	19	92.23%	103.94 u
^29^SiF_4_	29	19	4.67%	104.94 u
^30^SiF_4_	30	19	3.10%	105.93 u
^28^Si^1^H_4_	28	1	92.219(4)%	32.01 u
^28^Si^2^H_4_	28	2	0.010(6)%	36.03 u
^29^Si^1^H_4_	29	1	4.669(5)%	33.01 u
^29^Si^2^H_4_	29	2	0.000(5)%	37.03 u
^30^Si^1^H_4_	30	1	3.099(6)%	34.00 u
^30^Si^2^H_4_	30	2	0.000(4)%	38.14 u

**Table 3:** Technically significant differences in the physicochemical properties of natural silicon (^nat^Si) and isotopically pure silicon-28 (^28^Si).

	^nat^Si	^28^Si	Reference
Mass distribution	92.2%—28 u	100%—28 u	[102]
	4.7%—29 u		
	3.1%—30 u		
Thermal conductivity @ 21 K	45 Wcm^−1^K^−1^	450 Wcm^−1^K^−1^	[7]
Band gap	1.12 eV	1.06 eV	[8]
Nuclear spin	I = ^1^/_2_; Ī = ^√3^/_2_ ħ	I = 0; Ī = 0 ħ	

## Data Availability

No new data were generated for the purposes of this review. Should any queries arise regarding the data obtained from the literature, we would like to direct you to the original sources. Otherwise, the authors of this publication are also available to answer any questions that may arise.

## References

[1] Scappucci G., Taylor P.J., Williams J.R., Ginley T., Law S. (2021). Crystalline materials for quantum computing: Semiconductor heterostructures and topological insulators exemplars. MRS Bull..

[2] Zwerver A.M.J., Krähenmann T., Watson T.F., Lampert L., George H.C., Pillarisetty R., Bojarski S.A., Amin P., Amitonov S.V., Boter J.M. (2022). Qubits made by advanced semiconductor manufacturing. Nat. Electron..

[3] Itoh K.M., Watanabe H. (2014). Isotope engineering of silicon and diamond for quantum computing and sensing applications. MRS Commun..

[4] Inyushkin A.V., Taldenkov A.N., Gibin A.M., Gusev A.V., Pohl H.-J. (2004). On the isotope effect in thermal conductivity of silicon. Phys. Status Solidi.

[5] Kane B. (1998). A Silicon-based nuclear spin quantum computer. Nature.

[6] Liu Y., Rinner S., Remmele T., Ernst O., Reiserer A., Boeck T. (2022). 28Silicon-on-insulator for optically interfaced quantum emitters. J. Cryst. Growth.

[7] Inyushkin A.V., Taldenkov A.N., Ager J.W., Haller E.E., Riemann H., Abrosimov N.V., Pohl H.-J., Becker P. (2018). Ultrahigh thermal conductivity of isotopically enriched silicon. J. Appl. Phys..

[8] Karaiskaj D., Thewalt M.L.W., Ruf T., Cardona M., Pohl H.-J., Deviatych G.G., Sennikov P.G., Riemann H. (2001). Photoluminescence of Isotopically Purified Silicon: How Sharp are Bound Exciton Transitions?. Phys. Rev. Lett..

[9] Brooks M. (2019). Beyond quantum supremacy: The hunt for useful quantum computers. Nature.

[10] Boudon V., Richard C., Manceron L. (2022). High-Resolution spectroscopy and analysis of the fundamental modes of ^28^SiF_4_. Accurate experimental determination of the Si-F bond length. J. Mol. Spectrosc..

[11] Gordon J.P., Bowers K.D. (1958). Microwave spin echoes from donor electrons in Silicon. Phys. Rev. Lett..

[12] Sennikov P.G., Kornev R.A., Abrosimov N.V. (2015). Production of stable silicon and germanium isotopes via their enriched volatile compounds. J. Radioanal. Nucl. Chem..

[13] Kohen A., Limbach H.-H. (2006). Isotope Effects in Chemistry and Biology.

[14] Yu J. (2006). Production of silicon tetrafluoride (translated from Chinese). Wujiyan Gongye.

[15] Chen X.-H., Li Y., Zhang J.-G., Gao W.-L., Cao X., Chen Y., Liu Z.-D. (2015). Preparation and purification of silicon tetrafluoride (translated from Chinese). Guisuanyan Tongbao.

[16] Han J., Li X., Wang J. (2013). Research and development on process of silicon tetrafluoride and the progress thereof (translated from Chinese). Wujiyan Gongye.

[17] Loginov A.V., Garbar A.M. (1989). Silicon tetrafluoride. Properties, synthesis, and use (translated from Russian). Vysok. Veshchestva.

[18] Gelmboldt V.O., Ennan A.A. (2004). Silicon tetrafluoride: Prospects for its processing and use as chemical reagent (translated from Russian). Ekotek. I Resur..

[19] Davy H. (1812). An Account of Some Experiments on Different Combinations of Fluoric Acid. Philos. Trans. R. Soc. Lond..

[20] Jeffes J.H.E., Richardson F.D., Pearson J. (1954). The Heats of Formation of Manganous Orthosilicate and Manganous Sulphide. J. Trans. Faraday Soc..

[21] Joshi A.N. (2022). A review of processes for separation and utilization of fluorine from phosphoric acid and phosphate fertilizers. Chem. Pap..

[22] Ruscic B., Pinzon R.E., Morton M.L., von Laszewski G., Bittner S., Nijsure S.G., Amin K.A., Minkoff M., Wagner A.F. (2004). Introduction to Active Thermochemical Tables: Several “Key” Enthalpies of Formation Revisited. J. Phys. Chem. A.

[23] Padma D.K., Kalbandkeri R.G., Suresh B.S., Bhat V.S. (1991). Low temperature fluorination of some non-metals and non-metal compounds with fluorine. Indian J. Chem. Sect. A Inorg. Phys. Theor. Anal..

[24] Magomedbekov E.P., Chizhevskaya S.V., Davydov A.V., Zhukov A.V., Klimenko O.M., Sarychev G.A., Kudryavtsev E.M. (2012). Interaction of depleted uranium tetrafluoride with silica. At. Energy.

[25] Biehl E., Schubert U. (2000). Reaktionen von Siliciummonoxid mit Münzmetallhalogeniden. Monatsh. Chem..

[26] Bulanov A.D., Pryakhin D.A., Balabanov V.V. (2003). Preparation of High-Purity Silicon Tetrafluoride by Thermal Dissociation of Na2SiF6. Russ. J. Appl. Chem..

[27] Cankaya K., Pretzer W.R., Livingston W.A., Kana’an A.S. (1970). Equilibrium decomposition pressures of barium hexafluorosilicate. High Temp. Sci..

[28] Zachara J., Wisniewski W. (1995). Electronegativity force of cations and thermal decomposition of complex fluorides. I. Thermal decomposition of fluorosilicates. J. Therm. Anal..

[29] Krylov V.A., Sorochkina T.G., Bulanov A.D., Lashkov A.Y. (2012). C1-C4 hydrocarbon release in the preparation of SiF_4_ through Na_2_SiF_6_ pyrolysis. Inorg. Mater..

[30] Padma D.K., Kumar H.P.S. (1992). Displacement of Lewis acid gases—Phosphorus pentafluoride, boron fluoride, and silicon fluoride from their ammonium, alkali metal and pyridinium fluoro complexes by sulfur trioxide at room temperature. Synth. React. Inorg. Met.-Org. Chem..

[31] Padma D.K., Suresh B.S., Vasudevamurthy A.R. (1979). Silicon tetrafluoride: Preparation and reduction with lithium aluminum hydride. J. Fluor. Chem..

[32] Padma D.K., Murthy A.R. (1974). Vasudeva New method for the preparation of silicon tetrafluoride. J. Fluor. Chem..

[33] Roesky H.W., Herzog A., Keller K. (1994). Organotin fluorides as fluorinating reagents for chlorides of main group elements—Quantitative recycling of the fluorinating reagent. Z. Naturforsch. B J. Chem. Sci..

[34] Boehm H.P. (1969). Silicon tetrafluoride preparation. Z. Anorg. Allg. Chem..

[35] Lieser K.H., Rosenbaum I. (1967). Preparation of silicon tetrafluoride from silicon dioxide and lead fluoride. Z. Anorg. Allg. Chem..

[36] Stapf A., Gondek C., Kroke E., Roewer G., Yang D. (2018). Wafer Cleaning, Etching, and Texturization. Handbook of Photovoltaic Silicon.

[37] Gondek C., Hanich R., Honeit F., Lißner A., Stapf A., Kroke E. (2016). Etching Silicon with Aqueous Acidic Ozone Solutions: Reactivity Studies and Surface Investigations. J. Phys. Chem. C.

[38] Gondek C., Lippold M., Röver I., Bohmhammel K., Kroke E. (2014). Etching Silicon with HF-H_2_O_2_-based Mixtures: Reactivity Studies and Surface Investigations. J. Phys. Chem. C.

[39] Patzig-Klein S., Roewer G., Kroke E. (2010). New insights into acidic wet chemical silicon etching by HF/H_2_O-NOHSO_4_-H_2_SO_4_ solutions. Mater. Sci. Semicond. Process..

[40] Stapf A., Nattrodt P., Kroke E. (2018). On The Mechanism of the Anisotropic Dissolution of Silicon in Chlorine Containing Hydrofluoric Acid Solutions. J. Electrochem. Soc..

[41] Schubert N., Stapf A., Lißner A., Zomack N., Neumann A.-L., Kroke E. (2024). Analysis of silicon surfaces etched in aqueous HF-(HBr)–Br2-mixtures. Chem. Inorg. Mater..

[42] Dawei L., Xilu Y., Xianfeng Q., Yanan P., Xiaoyan L. (2023). Effects of HF Acid on Dissolution of Elemental Si. Silicon.

[43] Rietig A., Langner T., Acker J. (2019). A revised model of silicon oxidation during the dissolution of silicon in HF/HNO_3_ mixtures. Phys. Chem. Chem. Phys..

[44] Rietig A., Langner T., Acker J. (2022). Comprehensive stoichiometric studies on the reaction of silicon in HF/HNO_3_ and HF/HNO_3_/H_2_SiF_6_ mixtures. Phys. Chem. Chem. Phys..

[45] Haase M., Melzer M., Lang N., Ecke R., Zimmermann S., van Helden J.-P.H., Schulz S.E. (2020). On the relationship between SiF_4_ plasma species and sample properties in ultra low-k etching processes. AIP Adv..

[46] Osipov A.A., Iankevich G.A., Speshilova A.B., Osipov A.A., Endiiarova E.V., Berezenko V.I., Tyurikova I.A., Tyurikov K.S., Alexandrov S.E. (2020). High-temperature etching of SiC in SF_6_/O_2_ inductively coupled plasma. Sci. Rep..

[47] Tasaka A., Takahashi K., Tanaka K., Shimizu K., Mori K., Tada S., Shimizu S., Abe T., Inaba M., Ogumi Z. (2002). Plasma etching of SiC surface using NF_3_. J. Vac. Sci. Technol..

[48] Donnelly V.M., Kornblit A. (2013). Plasma Etching: Yesterday, Today, and Tomorrow. J. Vac. Sci. Technol. A.

[49] Saito Y., Yamaoka O., Yoshida A. (1990). Plasmaless cleaning process of silicon surface using chlorine trifluoride. Appl. Phys. Lett..

[50] Carver C.T., Plombon J.J., Romero P.E., Suri S., Tronic T.A., Turkot R.B. (2015). Atomic Layer Etching: An Industry Perspective. ECS J. Solid State Sci. Technol..

[51] Wu J., Zhang J., Cao Z., Liu Q., Wei F., Zhou J., Wang D., Shi S., Qian G. (2019). Improvement on Fluorine Migration from SF_6_ to SiF_4_ by an Efficient Mediator of Fe_2_O_3_/Cr_2_O_3_ Composites. ACS Appl. Mater. Interfaces.

[52] Natta G. (1930). Structure of silicon tetrafluoride. Gazz. Chim. Ital..

[53] Brockway L.O., Wall F.T. (1934). The Electron Diffraction Investigation of Some Non-metallic Halides. J. Am. Chem. Soc..

[54] Mootz D., Korte L. (1984). Fluoride und Fluorosäuren, VIII—Über eine fehlgeordnete feste Phase des SF_4_ sowie die Kristallstrukturen von Produkten seiner unbeabsichtigten Hydrolyse in Glasapparaturen, SiF_4_ (Neubestimmung) und SOF_2_. Z. Naturforsch..

[55] Schomaker V., Stevenson D.P. (1941). Some Revisions of the Covalent Radii and the Additivity Rule for the Lengths of Partially Ionic Single Covalent Bonds. J. Am. Chem. Soc..

[56] Pauling L. (1960). The Nature of the Chemical Bond.

[57] Gillespie R.J. (1998). Covalent and Ionic Molecules: Why Are BeF_2_ and AlF_3_ High Melting Point Solids whereas BF_3_ and SiF_4_ Are Gases?. J. Chem. Educ..

[58] Takami M., Kuze H. (1983). Infrared–microwave double resonance spectroscopy of the SiF_4_ ν3 fundamental using a tunable diode laser. J. Chem. Phys..

[59] Donald K.J., Böhm M.C., Lindner H.J. (2005). Analysis of competing bonding parameters. Part 2. The structure of halosilanes and halogermanes (MH_4−*n*_X*_n_*, *n* = 1–4; M=Si, Ge; X=F, Cl, Br). J. Mol. Struct. Theochem..

[60] Wang H., Wu P., Wu Z., Shi L., Cheng L. (2022). New insight into the electronic structure of SiF_4_: Synergistic back-donation and the eighteen-electron rule. Phys. Chem. Chem. Phys..

[61] Chamberlin A. (2016). Isotope Effects in Chemical Reactions. Annu. Rev. Phys. Chem..

[62] Otto R. (1931). Some physical constants of SiF_4_, WF_6_ and MoF_6_. Z. Anorg. Allg. Chem..

[63] Golovanov I.B. (2005). Quantitative Structure-Property Relationship: XXIV. Properties of Halo Derivatives of Methane, Silane, and Methylsilanes. Russ. J. Gen. Chem..

[64] Booth H.S., Swinehart C.F. (1935). Critical Constants and Vapor Pressure of Some Gaseous Fluorides of Group IV. J. Am. Chem. Soc..

[65] Hanson D.E., Santos C.M. (2022). Silicon Tetrafluoride Vapor Pressure Study. Technical Report. https://www.osti.gov/biblio/1908063/.

[66] Sukkaew P. (2016). Thermochemical Properties of Halides and Halohydrides of Silicon and Carbon. ECS J. Solid State Sci. Technol..

[67] Kickel B.L., Fisher E.R., Armentrout P.B. (1993). Dissociative charge-transfer reactions of atomic nitrogen (1+) (^3^P), dinitrogen (1+) (^2^Σ_g_^+^), argon(1+) (^2^P_3/2,1/2_), and krypton(1+) (^2^P_3/2_) with tetrafluorosilane. Thermochemistry of SiF_4_^+^ and SiF_3_^+^. J. Chem. Phys..

[68] Kolditz L. (1993). Anorganikum.

[69] McDonald J.D., Williams C.H., Thompson J.C., Margrave J.L. (1968). Appearance potentials, ionization potentials and heats of formation for perfluorosilanes and perfluoroborosilanes. Advan. Chem. Ser..

[70] Estrada-Alexanders A.F., Hurly J.J. (2008). Kinematic viscosity and speed of sound in gaseous CO, CO_2_, SiF_4_, SF_6_, C_4_F_8_, and NH_3_ from 220K to 375K and pressures up to 3.4 MPa. J. Chem. Thermodyn..

[71] Lide D.R. (1994). CRC Handbook of Chemistry and Physics.

[72] Nakamoto K. (2009). Infrared and Raman Spectra of Inorganic and Coordination Compounds: Part A: Theory and Applications in Inorganic Chemistry.

[73] Hirota E. (1985). High-Resolution Spectroscopy of Transient Molecules.

[74] McLafferty F.W., Tureček F. (1993). Interpretation of Mass Spectra.

[75] Levitt M.H. (2008). Spin Dynamics: Basics of Nuclear Magnetic Resonance.

[76] Claridge T.D.W. (2016). High-Resolution NMR Techniques in Organic Chemistry.

[77] Keeler J. (2010). Understanding NMR Spectroscopy.

[78] Lakowicz J.R. (2006). Principles of Fluorescence Spectroscopy.

[79] Atkins P., de Paula J. (2014). Physical Chemistry.

[80] Safety Data Sheet Silicon Tetrafluoride. https://produkte.linde-gas.at/sdb_konform/SiF4_10021730EN.pdf.

[81] Rom W.N., Markowitz S.B. (2006). Environmental and Occupational Medicine.

[82] Furr A.K. (2000). CRC Handbook of Laboratory Safety.

[83] Greenwood N.N., Earnshaw A. (1997). Chemistry of the Elements.

[84] Dobkin D.M., Zuraw M.K. (2003). Principles of Chemical Vapor Deposition.

[85] Wolf S., Tauber R.N. (2000). Silicon Processing for the VLSI Era. Process Technology.

[86] Rana T., Chandrashekhar M.V.S., Sudarshan T.S. (2012). Elimination of silicon gas phase nucleation using tetrafluorosilane (SiF_4_) precursor for high quality thick silicon carbide (SiC) homoepitaxy. Phys. Status Solidi A.

[87] Sennikov P., Pryakhin D., Andreev B., Gavrilenko L., Drozdov Y., Drozdov M., Pohl H.-J., Shashkin V. (2010). Plasma-enhanced chemical vapor deposition of 99.95% 28Si in form of nano- and polycrystals using silicon tetrafluoride precursor. Cryst. Res. Technol..

[88] Ingle W.M., Thompson S.W. (1979). Silicon purification process. US Patent.

[89] Becker M., Oles M. (1990). Silicon Tetrafluoride for the Synthesis of Ultra-pure Silicon. J. Cryst. Growth.

[90] Ryssel H., Ruge I. (1986). Ion Implantation: Equipment and Techniques.

[91] Sze S.M., Ng K.K. (2006). Physics of Semiconductor Devices.

[92] Smith J.D. (1965). Fluorination Reactions of Organic Compounds, Advances in Fluorine Chemistry.

[93] Wallace R., Kasap S., Capper P. (2006). Dielectric Materials for Microelectronics. Springer Handbook of Electronic and Photonic Materials.

[94] Grill A., Neumayer D.A. (2003). Structure of low dielectric constant to extreme low dielectric constant SiCOH films: Fourier tra nsform infrared spectroscopy characterization. J. Appl. Phys..

[95] Wolf S., Tauber R.N. (2002). Silicon Processing for the VLSI Era: Volume 4—Deep-Submicron Process Technology.

[96] Collins K.D., Smith D.K. (2008). Fluorosilicic Acid and its Industrial Applications. Ind. Eng. Chem. Res..

[97] Ullmann F., Gerhartz W., Yamamoto Y.S., Campbell F.T., Pfefferkorn R., Rounsaville J.F. (1986). Ullmann’s Encyclopedia of Industrial Chemistry.

[98] Corey E.J., Cheng X.M. (1995). The Logic of Chemical Synthesis.

[99] Ojima I. (2010). Catalytic Asymmetric Synthesis.

[100] Wagler J., Böhme U., Kroke E. (2014). Higher-Coordinated Molecular Silicon Compounds. Funct. Mol. Silicon Compd. I Regul. Oxid. States.

[101] Elschenbroich C. (2006). Organometallics.

[102] Hollemann A.F., Wiberg E. (1995). Lehrbuch der Anorganischen Chemie.

[103] Levin E.V. (1994). Separation of multicomponent isotopic mixtures in a gas centrifuge—Approximate method for solving the system of diffusion-transport equations and analysis of some separation characteristics. At. Energy.

[104] Borisevich V., Levin E. (2001). Separation of Multicomponent Isotope Mixtures by Gas Centrifuge. Sep. Sci. Technol..

[105] URENCO Limited (2019). Information der Öffentlichkeit nach der Strahlenschutzverordnung und der Störfallverordnung. https://www.urenco.com/cdn/uploads/supporting-files/UD_Information_booklet_260623.pdf.

[106] https://www.internetchemistry.com.

[107] Glaser A. (2008). Characteristics of the Gas Centrifuge for Uranium Enrichment and Their Relevance for Nuclear Weapon Proliferation. Sci. Glob. Secur..

[108] Kushner D. (2013). The Real Story of Stuxnet. IEEE Spectr..

[109] Bogovalov S.V., Borman V.D. (2015). Isotope Separation in Concurrent Gas Centrifuges. Phys. Procedia.

[110] Snyder R. (2016). A Proliferation Assessment of Third Generation Laser Uranium Enrichment Technology. Sci. Glob. Secur..

[111] Bokhan P.A., Buchanov V.V., Fateev N.V., Kalugin M.M., Kazaryan M.A., Prokhorov A.M., Zakrevskii D.E. (2006). Laser Isotope Separation in Atomic Vapor.

[112] Eerkens J.W. (1976). Spectral considerations in the laser isotope separation of Uranium Hexafluoride. Appl. Phys..

[113] Baranov I.Y., Koptev A.V. (2010). Mode-Locked CO Laser for Isotope Separation of Uranium Employing Condensation Repression. Adv. Opt. Technol..

[114] Nundy U., Kumar M. (2012). Generation of tunable 16 μm radiation from CO_2_ by cascade lasing. Pramana J. Phys..

[115] Li D.J., Yang G.L., Chen F., Xie J.J., Zhang L.M., Guo J., Shao C.L., Peng Z.Q., Lu Q.P. (2012). Stimulated rotational Raman scattering at multiwavelength under tea CO_2_ laser pumping with a multiple-pass cell. Laser Phys..

[116] https://www.osti.gov/etdeweb/biblio/20073948.

[117] https://www.silex.com.au/silex-technology/silex-zs-si-production-for-quantum-computing/.

[118] https://www.silex.com.au/investors/announcements/.

[119] Dargan J. (2023). Quantum Silicon Production Project Awarded $5.1M Funding under the Defence Trailblazer Program. Quantum Insid..

[120] Miller D.G. (1960). Thermodynamics of Irreversible Processes. The Experimental Verification of the Onsager Reciprocal Relations. Chem. Rev..

[121] Rowe D.M. (2018). Thermoelectrics Handbook.

[122] Welty J.R., Wicks C.E., Wilson R.E., Rorrer G.L. (2008). Fundamentals of Momentum, Heat and Mass Transfer.

[123] Cotton S. (2006). Lanthanide and actinide chemistry.

[124] Liehr G., Die AKW-Krokodile (1997). Taz. https://taz.de/Die-AKW-Krokodile/!1380786/.

[125] Musik A., Die Krokodile von Tricastin (2011). Diepresse. https://www.diepresse.com/643109/die-krokodile-von-tricastin.

[126] Hiltzik M.A. (2015). Big Science: Ernest Lawrence and the Invention that Launched the Military-Industrial Complex.

[127] Love L.O. (1979). Electromagnetic Separation of Isotopes at Oak Ridge. Science.

[128] Clarence L. (2003). The Role of Chemistry in the Oak Ridge Electromagnetic Project. Bull. Hist. Chem..

[129] Albright D., Hibbs M. (1991). Iraq’s Nuclear Hide-and-Seek. Bull. At. Sci..

[130] Gsponer A., Hurni J.-P. (1995). Iraq’s Calutrons Electromagnetic Isotope Separation, Beam Technology and Nuclear Weapon Proliferation.

[131] Tang K., Kim H.S., Ramanayaka A.N.R., Simons D.S., Pomeroy J.M. (2019). A compact, ultra-high vacuum ion source for isotopically enriching and depositing ^28^Si thin films. Rev. Sci. Instrum..

[132] Tang K., Kim H.S., Ramanayaka A.N.R., Simons D.S., Pomeroy J.M. (2020). Targeted enrichment of ^28^Si thin films for quantum computing. J. Phys. Commun..

[133] Holmes D., Johnson B.C., Chua C., Voisin B., Kocsis S., Rubanov S., Robson S.G., McCallum J.C., McCamey D.R., Rogge S. (2021). Isotopic enrichment of silicon by high fluence ^28^Si^−^ ion implantation. Phys. Rev. Mater..

[134] Acharya R., Coke M., Adshead M., Li K., Achinuq B., Cai R., Gholizadeh A.B., Jacobs J., Boland J.L., Haigh S.J. (2024). Highly ^28^Si enriched silicon by localised focused ion beam implantation. Commun. Mater..

[135] Becker E.W., Bley P., Ehrfeld W., Lenné H. (1978). Uranisotopentrennung mit einem Gegenstromwirbelrohr. Z. Naturforsch. A Phys. Sci..

[136] Becker E.W., Bier W., Bley P., Ehrfeld U., Ehrfeld W., Eisenbeiß G. (1979). Das Entwicklungspotential des Trenndüsenverfahrens zur U-235-Anreicherung. Atomwirtschaft.

[137] Becker E.W., Bier W., Ehrfeld W., Schubert K., Seidel D. (1981). Entwicklung und technische Anwendung des Trenndüsenver-fahrens zur Anreicherung von Uran 235. KfK-Nachrichten.

[138] Albright D. (1994). South Africa’s Secret Nuclear Weapons (ISIS Reports). https://www.isis-online.org/publications/southafrica/ir0594.html.

[139] Cochran T.B. (1994). Highly Enriched Uranium Production for South African Nuclear Weapons. Sci. Glob. Secur..

[140] Becker P., Pohl H.-J., Riemann H., Abrosimov N. (2010). Enrichment of silicon for a better kilogram. Phys. Status Solidi A.

[141] Pfann W.G. (1952). Principles of Zone-Melting. JOM.

[142] Zakharkin L.I., Gavrilenko V.V., Khorlina I.M., Zhigareva G.G. (1962). The reduction of silicon and germanium chlorides and alkoxides by sodium and potassium aluminum hydrides. Bull. Acad. Sci. USSR Div. Chem. Sci. (Engl. Transl.).

[143] Lefrancois P.A. (1982). Production of Silane.

[144] Devyatykh G.G., Dianov E.M., Bulanov A.D., Troshin O.Y., Balabanov V.V., Pryakhin D.A. (2003). Preparation of high-purity monoisotopic silane: ^28^SiH_4_, ^29^SiH_4_, and ^30^SiH_4_. Dokl. Chem..

[145] Bulanov A.D., Mikheev V.S., Troshin O.Y., Lashkov A.Y. (2008). Reaction of Silicon Tetrafluoride with Calcium Hydride as a Propagating Wave. Russ. J. Inorg. Chem..

[146] Troshin O.Y., Bulanov A.D., Mikheev V.S., Lashkov A.Y. (2010). Mechanically Activated Synthesis of Monosilane by the Reaction of Calcium Hydride with Silicon Tetrafluoride. Russ. J. Inorg. Chem..

[147] Fadeev L.L., Kvaratskheli J.K., Zhirkov M.S., Ivashin A.M., Kudrjavtsev V.V., Grishin A.V., Filipinov V.T. (1997). Method of preparing monosilane.

[148] Churbanov M.F., Bulanov A.D., Kotkov A.P., Potapov A.M., Troshin O.Y., Lashkov A.Y., Grishnova N.D., Adamchik S.A. (2010). Production of Silanes ^29^SiH_4_ and ^30^SiH_4_ of High Chemical and Isotopic Purity. Dokl. Chem..

[149] Sennikov P.G., Kotkov A.P., Adamchik S.A., Grishnova N.D., Chuprov L.A., Ignatov S.A. (2010). Impurities in Monosilanes Synthesized by Different Processes. Inorg. Mater..

[150] Bulanov A.D., Balabanov V.V., Pryakhin D.A., Troshin O.Y. (2002). Preparation and Fine Purification of SiF_4_ and ^28^SiH_4_. Inorg. Mater..

[151] Ager III J.W., Beeman J.W., Hansen W.L., Haller E.E., Sharp I.D., Liao C., Yang A., Thewalt M.L.W., Riemann H. (2005). High-Purity, Isotopically Enriched Bulk Silicon. J. Electrochem. Soc..

[152] Weerts W.L.M., de Croon M.H.J.M., Martin G.B. (1998). The Kinetics of the Low-Pressure Chemical Vapor Deposition of Polycrystalline Silicon from Silane. J. Electrochem. Soc..

[153] Becker P., Friedrich H., Fujii K., Giardini W., Mana G., Picard A., Pohl H.-J., Riemann H., Valkiers S. (2009). The Avogadro constant determination via enriched silicon-28. Meas. Sci. Technol..

[154] Mazzocchi V., Sennikov P.G., Bulanov A.D., Churbanov M.F., Bertrand B., Hutin L., Barnes J.P., Drozdov M.N., Hartmann J.M., Sanquer M. (2019). 99.992% ^28^Si CVD-grown epilayer on 300 mm substrates for large scale integration of silicon spin qubits. J. Cryst. Growth.

[155] Baptista A., Silva F., Porteiro J., Míguez J., Pinto G. (2018). Sputtering Physical Vapor Deposition (PVT) Coatings: A Critical Review on Process Improvement and Market Trend Demands. Coatings.

[156] Bean J.C. (1984). Silicon MBE: From strained-layer epitaxy to device application. J. Cryst. Growth.

[157] Ramanayaka A.N., Tang K., Hagmann J.A., Kim H.-S., Simons D.S., Richter C.A., Pomeroy J.M. (2019). Use of quantum effects as potential qualifying metrics for “quantum grade silicon”. AIP Adv..

[158] Andreas B., Azuma Y., Bartl G., Becker P., Bettin H., Borys M., Busch I., Gray M., Fuchs P., Fujii K. (2011). Determination of the Avogadro Constant by Counting the Atoms in a ^28^Si Crystal. Phys. Rev. Lett..

[159] https://www.wissenschaft.de/erde-umwelt/kampf-ums-kilo/.

[160] https://www.britannica.com/science/metric-system-measurement#ref258582.

[161] https://www.spiegel.de/wissenschaft/mensch/physikalische-masseinheiten-das-raetselhafte-schrumpfen-des-urkilogramms-a-505526.html.

[162] Girard G. (1994). The Third Periodic Verification of National Prototypes of the Kilogram (1988–1992). Metrologia.

[163] https://leopard.tu-braunschweig.de/receive/dbbs_mods_00032342.

[164] https://www.nytimes.com/2018/11/16/science/kilogram-physics-measurement.html.

[165] https://web.archive.org/web/20210402142630/https://www.bipm.org/utils/en/pdf/CGPM/Draft-Resolution-A-EN.pdf.

[166] Robinson I.A., Schlamminger S. (2016). The watt or Kibble balance: A technique for implementing the new SI definition of the unit of mass. Metrologia.

[167] Azuma Y., Barat P., Bartl G., Bettin H., Borys M., Busch I., Cibik L., D’Agostino G., Fujii K., Fujimoto H. (2015). Improved measurement results for the Avogadro constant using a ^28^Si-enriched crystal. Metrologia.

[168] Andreas B., Azuma Y., Bartl G., Becker P., Bettin H., Borys M., Busch I., Fuchs P., Fujii K., Fujimoto H. (2011). Counting the atoms in a ^28^Si crystal for a new kilogram definition. Metrologia.

[169] Bulska E., Drozdov M.N., Mana G., Pramann A., Rienitz O., Sennikov P., Valkiers S. (2011). The isotopic composition of enriched Si: A data analysis. Metrologia.

[170] Picard A., Barat P., Borys M., Firlus M., Mizushima S. (2011). State-of-the-art mass determination of ^28^Si spheres for the Avogadro project. Metrologia.

[171] Pramann A., Rienitz O. (2016). Mass Spectrometric Investigation of Silicon Extremely Enriched in ^28^Si: From ^28^SiF_4_ (Gas Phase IRMS) to ^28^Si Crystals (MC-ICP-MS). Anal. Chem..

[172] Pramann A., Lee K.-S., Noordmann J., Rienitz O. (2015). Probing the homogeneity of the isotopic composition and molar mass of the ‘Avogadro’-crystal. Metrologia.

[173] Pramann A., Rienitz O., Noordmann J., Güttler B., Schiel D. (2014). A More Accurate Molar Mass of Silicon via High Resolution MC-ICP-Mass Spectrometry. Z. Phys. Chem..

[174] Rienitz O., Pramann A., Schiel D. (2010). Novel concept for the mass spectrometric determination of absolute isotopic abundances with improved measurement uncertainty: Part 1—Theoretical derivation and feasibility study. Int. J. Mass Spectrom..

[175] Pramann A., Rienitz O., Schiel D., Güttler B. (2011). Novel concept for the mass spectrometric determination of absolute isotopic abundances with improved measurement uncertainty: Part 2—Development of an experimental procedure for the determination of the molar mass of silicon using MC-ICP-MS. Int. J. Mass Spectrom..

[176] Pramann A., Rienitz O., Schiel D., Güttler B., Valkiers S. (2011). Novel concept for the mass spectrometric determination of absolute isotopic abundances with improved measurement uncertainty: Part 3—Molar mass of silicon highly enriched in ^28^Si. Int. J. Mass Spectrom..

[177] Haller E.E. (1995). Isotopically engineered semiconductors. J. Appl. Phys..

[178] Yamaguchi H., Riveros R.E., Mitsuishi I., Takagi U., Ezoe Y., Yamasaki N., Mitsuda K., Hashimoto F. (2010). Magnetic field-assisted finishing for micropore X-ray focusing mirrors fabricated by deep reactive ion etching. CIRP Ann..

[179] Li Q., Raimbault V., Calmon P.-F., Reig B., Debernardi P., Ottevaere H., Doucet J.-B., Roul J., Bardinal V. (2023). Direct 3D-printing of microlens on single mode polarization-stable VCSEL chip for miniaturized optical spectroscopy. J. Opt. Microsyst..

[180] Shvyd’ko Y. (2004). X-ray Optics: High-Energy-Resolution Applications (Springer Series in Optical Sciences, 98).

[181] Hawking S., Hawking S.W., Israel W. (1987). Three Hundred Years of Gravitation.

[182] Abbott B.P., Abbott R., Abbott T.D., Abernathy M.R., Acernese F., Ackley K., Adams C., Adams T., Addesso P., Adhikari R.X. (2016). Observation of Gravitational Waves from a Binary Black Hole Merger. Phys. Rev. Lett..

[183] Hild S., Chelkowski S., Freise A. (2008). Pushing towards the ET sensitivity using ‘conventional’ technology. arXiv.

[184] https://web.archive.org/web/20170620143851/https://tds.virgo-gw.eu/?call_file=ET-0106C-10.pdf.

[185] Di Giovanni M., Giunchi C., Saccorotti G., Berbellini A., Boschi L., Olivieri M., De Rosa R., Naticchioni L., Oggiano G., Carpinelli M. (2020). A Seismological Study of the Sos Enattos Area—The Sardinia Candidate Site for the Einstein Telescope. Seismol. Res. Lett..

[186] https://www.einsteintelescope-emr.eu/de/2024/02/21/der-prototyp-des-einstein-teleskops-e-test-besteht-seine-erste-testreihe/.

[187] https://www.ikz-berlin.de/en/cosmology-particle-physics.

[188] https://www.einstein-teleskop.de/partner/.

[189] https://www.deutscheszentrumastrophysik.de.

[190] https://www.esa.int/Science_Exploration/Space_Science/LISA/Capturing_the_ripples_of_spacetime_LISA_gets_go-ahead.

[191] Ernst O.C., Liu Y., Boeck T. (2022). Leveraging dewetting models rather than nucleation models: Current crystallographic challenges in interfacial and nanomaterials research. Z. Kristallogr. Cryst. Mater..

[192] Schreiber L.R., Bluhm H. (2018). Toward a silicon-based quantum computer. Science.

[193] Almudever C.G., Lao L., Fu X., Khammassi N., Ashraf I., Iorga D., Varsamopoulos S., Eichler C., Wallraff A., Geck L. (2017). The Engineering Challenges in Quantum Computing.

[194] Loss D., DiVincenzo D.P. (1998). Quantum computation with quantum dots. Phys. Rev. A.

[195] Vandersypen L.M.K., Eriksson M.A. (2019). Quantum computing with semiconductor spins. Phys. Today.

[196] Zwanenburg F.A., Dzurak A.S., Morello A., Simmons M.Y., Hollenberg L.C.L., Klimeck G., Rogge S., Coppersmith S.N., Eriksson M.A. (2013). Silicon quantum electronics. Rev. Mod. Phys..

[197] Gritsch A., Ulanowski A., Reiserer A. (2023). Purcell enhancement of sing le-photon emitters in silicon. Optica.

[198] Johnston A., Felix-Rendon U., Wong Y.-E., Chen S. (2024). Cavity-coupled telecom atomic source in silicon. Nat. Commun..

[199] Zhang X., Li H.-O., Cao G., Xiao M., Guo G.-C., Guo G.-P. (2019). Semiconductor quantum computation. Natl. Sci. Rev..

[200] Jia Z., Fu Y., Cao Z., Cheng W., Zhao Y., Dou M., Duan P., Kong W., Cao G., Li H. (2022). Superconducting and Silicon-Based Semiconductor Quantum Computers: A review. IEEE Nanotechnol. Mag..

[201] Bluhm H., Schreiber L.R. Semiconductor Spin Qubits—A S calable Platform for Quantum Computing?. Proceedings of the 2019 IEEE International Symposium on Circuits and Systems (ISCAS).

[202] Schreiber L., Bluhm H. (2014). Silicon comes back. Nat. Nanotech..

[203] Laucht A., Hohls F., Ubbelohde N., Gonzalez-Zalba M.F., Reilly D.J., Stobbe S., Schröder T., Scarlino P., Koski J.V., Dzurak A. (2021). Roadmap on quantum nanotechnologies. Nanotechnology.

[204] Sabbagh D., Thomas N., Torres J., Pillarisetty R., Amin P., George H., Singh K., Budrevich A., Robinson M., Merrill D. (2019). Quantum Transport Properties of Industrial ^28^Si/^28^SiO_2_. Phys. Rev. Appl..

[205] Xue X., Patra B., van Dijk J.P.G., Samkharadze N., Subramanian S., Corna A., Wuetz B.P., Jeon C., Sheikh F., Juarez-Hernandez E. (2021). CMOS-based cryogenic control of silicon quantum circuits. Nature.

[206] Shankar S., Tyryshkin A.M., He J., Lyon S.A. (2010). Spin relaxation and coherence times for electrons at the Si/SiO_2_ interface. Phys. Rev. B.

[207] Schäffler F. (1997). High-mobility Si and Ge structures. Semicond. Sci. Technol..

[208] Watson T.F., Philips S.G.J., Kawakami E., Ward D.R., Scarlino P., Veldhorst M., Savage D.E., Lagally M.G., Friesen M., Coppersmith S.N. (2018). A programmable two-qubit quantum processor in silicon. Nature.

[209] Hollmann A., Struck T., Langrock V., Schmidbauer A., Schauer F., Leonhardt T., Sawano K., Riemann H., Abrosimov N.V., Bougeard D. (2020). Large, Tunable Valley Splitting and Single-Spin Relaxation Mechanisms in a Si/Si_*x*_Ge_1−*x*_ Quantum Dot. Phys. Rev. Appl..

[210] Simmons S., Brown R.M., Riemann H., Abrosimov N.V., Becker P., Pohl H.-J., Thewalt M.L.W., Itoh K.M., Morton J.J.L. (2011). Entanglement in a solid-state spin ensemble. Nature.

[211] Liu Y., Gradwohl K.-P., Lu C.-H., Yamamoto Y., Remmele T., Corley-Wiciak C., Teubner T., Richter C., Albrecht M., Boeck T. (2023). Growth of ^28^Si Quantum Well Layers for Qubits by a Hybrid MBE/CVD Technique. ECS J. Solid State Sci. Technol..

[212] Scappucci G., Kloeffel C., Zwanenburg F.A., Loss D., Myronov M., Zhang J.-J., De Franceschi S., Katsaros G., Veldhorst M. (2021). The germanium quantum information route. Nat. Rev. Mater..

[213] Hill C.D., Peretz E., Hile S.J., House M.G., Fuechsle M., Rogge S., Simmons M.Y., Hollenberg L.C.L. (2015). A surface code quantum computer in silicon. Sci. Adv..

[214] Morton J.J.L., Tyryshkin A.M., Brown R.M., Shankar S., Lovett B.W., Ardavan A., Schenkel T., Haller E.E., Ager J.W., Lyon S.A. (2008). Solid-state quantum memory using the ^31^P nuclear spin. Nature.

[215] O’Brien J.L., Schofield S.R., Simmons M.Y., Clark R.G., Dzurak A.S., Curson N.J., Kane B.E., McAlpine N.S., Hawley M.E., Brown G.W. (2001). Towards the fabrication of phosphorus qubits for a silicon quantum computer. Phys. Rev. B.

[216] Parthasarathy S.K., Kallinger B., Kaiser F., Berwian P., Dasari D.B.R., Friedrich J., Nagy R. (2023). Scalable Quantum Memory Nodes Using Nuclear Spins in Silicon Carbide. Phys. Rev. Appl..

[217] Babin C., Stöhr R., Morioka N., Linkewitz T., Steidl T., Wörnle R., Liu D., Hesselmeier E., Vorobyov V., Denisenko A. (2022). Fabrication and nanophotonic waveguide integration of silicon carbide colour centres with preserved spin-optical coherence. Nat. Mater..

[218] Plotnichenko V.G., Nazaryants V.O., Kryukova E.B., Koltashev V.V., Sokolov V.O., Gusev A.V., Gavva V.A., Kotereva T.V., Churbanov M.F., Dianov E.M. (2011). Refractive index spectral dependence, Raman spectra, and transmission spectra of high-purity ^28^Si, ^29^Si, ^30^Si, and ^nat^Si single crystals. Appl. Opt..

[219] Moutanabbir O., Senz S., Zhang Z., Gösele U. (2009). Synthesis of isotopically controlled metal-catalyzed silicon nanowires. Nano Today.

[220] Wagner R.S., Ellis W.C. (1964). Vapor-Liquid-Solid mechanism of Single-Crystal growth. Appl. Phys. Lett..

[221] Ernst O.C., Uebel D., Kayser S., Lange F., Teubner T., Boeck T. (2021). Revealing all states of dewetting of a thin gold layer on a silicon surface by nanosecond laser conditioning. Appl. Surf. Sci. Adv..

[222] Ernst O.C., Lange F., Uebel D., Teubner T., Boeck T. (2020). Analysis of catalyst surface wetting: The early stage of epitaxial germanium nanowire growth. Beilstein J. Nanotechnol..

[223] Lange F., Ernst O., Teubner T., Richter C., Schmidbauer M., Skibitzki O., Schroeder T., Schmidt P., Boeck T. (2020). In-plane growth of germanium nanowires on nanostructured Si(001)/SiO_2_ substrates. Nano Futures.

[224] Shi L., Yao D., Zhang G., Li B. (2009). Size dependent thermoelectric properties of silicon nanowires. Appl. Phys. Lett..

[225] Ci P., Sun M., Upadhyaya M., Song H., Jin L., Sun B., Jones M.R., Ager J.W., Aksamija Z., Wu J. (2022). Giant Isotope Effect of Thermal Conductivity in Silicon Nanowires. Phys. Rev. Lett..

[226] http://isosilicon.com/wp-content/uploads/2016/01/JO-Odden-Si-28.pdf.

[227] Nobuyoshi F., Satoshi U., Hirotoshi Y. (2005). Monoisotopic Silicon-28 (28Si). Japanese Patent.

[228] Fujimaki N., Ushio S. (2005). Solar Cell And Method For Manufacturing The Same. Japanese Patent.

[229] https://gepris.dfg.de/gepris/projekt/221263527?context=projekt&task=showDetail&id=221263527&.

[230] https://prosjektbanken.forskningsradet.no/en/project/FORISS/193329?Kilde=FORISS&distribution=Ar&chart=bar&calcType=funding&Sprak=no&sortBy=score&sortOrder=desc&resultCount=30&offset=90&Fritekst=INVENT&source=FORISS&projectId=176869.

